# Spatial analysis of ecosystem service relationships to improve targeting of payments for hydrological services

**DOI:** 10.1371/journal.pone.0192560

**Published:** 2018-02-20

**Authors:** Pierre Mokondoko, Robert H. Manson, Taylor H. Ricketts, Daniel Geissert

**Affiliations:** 1 Postgraduate Division, Instituto de Ecología A.C., Xalapa, Veracruz, México; 2 Functional Ecology Netwrok, Instituto de Ecología A.C., Xalapa, Veracruz, México; 3 Rubenstein School for Environment and Natural Resources, University of Vermont, Burlington, Vermont, United States of America; 4 Gund Institute for Environment, University of Vermont, Burlington, Vermont, United States of America; Kerala Forest Research Institute, INDIA

## Abstract

Payment for hydrological services (PHS) are popular tools for conserving ecosystems and their water-related services. However, improving the spatial targeting and impacts of PHS, as well as their ability to foster synergies with other ecosystem services (ES), remain challenging. We aimed at using spatial analyses to evaluate the targeting performance of México’s National PHS program in central Veracruz. We quantified the effectiveness of areas targeted for PHS in actually covering areas of high HS provision and social priority during 2003–2013. First, we quantified provisioning and spatial distributions of two target (water yield and soil retention), and one non-target ES (carbon storage) using InVEST. Subsequently, pairwise relationships among ES were quantified by using spatial correlation and overlap analyses. Finally, we evaluated targeting by: (i) prioritizing areas of individual and overlapping ES; (ii) quantifying spatial co-occurrences of these priority areas with those targeted by PHS; (iii) evaluating the extent to which PHS directly contribute to HS delivery; and (iv), testing if PHS targeted areas disproportionately covered areas with high ecological and social priority. We found that modelled priority areas exhibited non-random distributions and distinct spatial patterns. Our results show significant pairwise correlations between all ES suggesting synergistic relationships. However, our analysis showed a significantly lower overlap than expected and thus significant mismatches between PHS targeted areas and all types of priority areas. These findings suggest that the targeting of areas with high HS provisioning and social priority by Mexico’s PHS program could be improved significantly. This study underscores: (1) the importance of using maps of HS provisioning as main targeting criteria in PHS design to channel payments towards areas that require future conservation, and (2) the need for future research that helps balance ecological and socioeconomic targeting criteria.

## Introduction

The capacity of ecosystems to provide multiple ecosystem services (ES) is recognized in both environmental science and public policy [1–2]. ES are produced by a combination of ecological processes [3–4] that provide both tangible and intangible benefits to human well-being [5]. Global ES supply is nevertheless declining and increasingly threatened by the overexploitation of ecosystems [6]. Consequently, research on ES has grown in recent years focusing in particular on the factors influencing the magnitude and spatial patterns of service provisioning [7–9]. Decision-makers in turn are increasingly using the ES concept as a cornerstone for the design of policy and economic instruments to support ecosystem conservation [10–11]. Such instruments are viewed as promising planning tools to optimize multiple benefits such as promoting sustainable development and improving management practices [12–13].

Payments for Ecosystem Services (PES) programs are increasingly popular policy instruments for promoting ES conservation [14–16]. Many countries have experimented with these programs [17–18] to target a variety of ES including water-related services, carbon sequestration, and biodiversity [19–20]. However, most have focused on hydrological services (HS). Since water scarcity and contamination are serious problems worldwide [21–22], restoring HS is fundamental for sustaining regional economic activities [23–24]. Payments for Hydrological Services (PHS) are of particular interest in Mexico, where HS degradation is increasingly acute [25–26].

Mexico’s national PHS program was created and is operated by the National Forest Commission (CONAFOR). Since 2003, CONAFOR pays rural landowners to conserve forests within overexploited watersheds to support HS provision, while supporting poverty alleviation [27–29]. However, this program uses a complex and evolving set of targeting criteria that are increasingly skewed towards social factors that could reduce program effectiveness [22,30]. Therefore, one unresolved issue is the need for scientific data to improve their spatial targeting. Although, this program aims to reduce soil erosion and improve water quality and supply, such HS are not directly monitored [28,31]. Instead, relying on the largely untested assumption of avoided deforestation serving as a proxy for the enhancement of HS [31–33]. As Wunder et al. [34] notes, for any payment to be conditional it must be possible to verify the existing service provisioning. If PHS are not conditional and have inadequate baselines [27–28], their ability to effectively conserve important areas for HS could be undermined and under-provision is a likely result. However, to our knowledge conditionality and the use of spatial distribution of HS as restrictive criteria have been overlooked to date.

An increasing number of studies have questioned the extent to which PHS programs effectively ensure provisioning of targeted services [22,35–36]. Various factors have been identified that should be addressed to improve program effectiveness. These include: (i) the assumption of linear relationships between forests and ES supply, with little supporting evidence [37–38]; (ii) poor understanding of synergies among bundled ES (e.g. set of ES that appear together repeatedly) and their spatial distributions [8,20,39]; (iii) the narrow focus on target services that limits the staking of ES (e.g. when multiple overlapping ES are sold separately to compensate for different impacts) and the identification of potential users who could help finance programs [12,26,40–41]; and finally, (iv) difficulties in quantifying program impacts on service supply due to a lack of direct monitoring [42–43].

Spatially explicit tools for quantifying and mapping ES can help to address these concerns [43]. Examples include assessing the factors influencing HS distribution, prioritizing areas of provision, and quantifying trade-offs or synergies between target and non-target HS [44]. These tools also guide decisions about where investments will yield maximum benefits and can gain momentum in policy realms [9,45]. A range of GIS-based tools have been used to map HS and facilitates the analysis of their magnitude and spatial relationships [46–47]. One open-source tool that offers standardized methods to do this and that has seen increased use in the ES literature is InVEST (Integrated Valuation of Ecosystem Services and Tradeoffs; [48]). InVEST has been further enhanced to allow simulations of how land use/land cover (LULC) might contribute to HS provisioning [13]. However, in the context of PHS its use has been limited and largely focused on exploring the spatial match between protected areas and service provisioning [49–50]. So far, few studies have directly applied mapping tools to assess spatial targeting and thus evaluate how PHS programs could maximize their ecological impacts [34].

More quantitative evaluations of how PHS programs succeed or fail in achieving their objectives are urgently needed [35]. Relatively, few programs have been subject to evaluations of their effectiveness and impacts [23,27,32–33]. Research has tended to focus narrowly on quantifying the impact of PHS in reducing poverty or deforestation rather than ensuring provision of HS to downstream users [51–52]. As relationships between poverty and PHS are complex, an animated discussion about their trade-offs is ongoing in the literature. Shapiro-Garza [31] explained how these competing agendas have been clashing to influence Mexico’s PHS program. CONAFOR conceived the PHS as mechanisms to primarily target environmental purposes [52]. However, interactions with other stakeholders have resulted in the hybridization of this program, targeting also threatened forests and areas of high marginalization [31,52]. Since HS and marginalization levels are heterogeneously distributed [36,51] the capacity of the program to cover both targets depends on its ability to optimize the distribution and impact of payments [18–19,47]. However, improvements in choosing which forests should be prioritized for PHS remains largely understudied.

Targeting is typically accomplished using a mix of spatially explicit geo-physical and socio-economic data [53–55]. Mexico’s PHS program is no exception with operators determining eligible zones using both types of data as targeting and selection ranking criteria, respectively [27,56]. Initially (2003–2006) eligible zones were required to be within hydrological vulnerable areas, in the buffer zones of protected areas, upstream from urban centers, or within priority mountainous areas [31–33]. Preference was given to parcels with more forest cover (>80%) [52]. Following criticism of this process, in 2007 CONAFOR introduced a scoring system privileging some lower density forest types [51]. Emphasis was placed on payments within areas with high risk of deforestation and elevated economic and social marginalization [54]. Introduction of social criteria (e.g. marginalization index, percentage of indigenous population, among others) progressively increased the number of criteria from nine in 2006 to 26 in 2010 [30]. However, resulting in a net decline of the influence of biophysical factors in the total ranking score. Some authors express concern about this trend away from ecological to social aims that may lead to possible losses in program effectiveness [56–57].

Attempts to evaluate targeting performance of PHS, in Mexico and elsewhere, rarely use criteria directly related to HS provisioning [22,58]. Instead relying on indirect measures of land use/land cover and the impact on forest cover to justify water provisioning [32–33,58]. However, few studies have paid attention on how PHS directly or indirectly contribute to HS delivery. Studies, primarily from economists, using poverty and deforestation metrics as evaluation criteria have yielded mixed results. Some show relatively low additionality and no evidence of a strong equity in enrolling forests that address both ecological and social concerns [23,51,59–60]. Additionality refers to the amount of avoided deforestation achieved with PHS compared to the amount of deforestation without payments [31]. Others studies suggest that the Mexico’s PHS program has successfully combined both goals [30–32,56]. Nevertheless, largely absent from this debate are criteria for evaluating PHS effectiveness in actually helping to insure HS provisioning. This is particularly important, because some studies have questioned the assumption that increased forest cover enhance provisioning of HS [33]. While forests are typically the main target of PHS, key drivers of HS are more sensitive to climate factors (e.g. temperature, humidity, cloud cover, and precipitation) [21,61–62]. Is therefore important to consider spatial heterogeneity of HS in the targeting of PHS [18,24]. Spatial analyses and HS maps may help to address such concerns by prioritizing areas for HS and clarifying their relationships with different LULC types [56,63]. Such knowledge may also allows targeting in areas to ensure conditionality for downstream user and strengthen these PHS [12,49,62].

Beyond spatial targeting for individual HS, mapping efforts allow to analyze spatial relationships with non-target ES to increase interest of different stakeholders [41,64–65]. However, since ES interact in complex ways and operate at particular scales, their evaluation remains challenging [12,66]. Understanding spatial co-occurrences, synergies, and trade-offs among multiple ES also remains limited [67–68]. Recent studies mapping ES co-occurrences have typically used correlation and overlap analyses to explore ES relationships and identify locations that optimize their simultaneous provision [11,39,49]. Naidoo and Ricketts [7], and most subsequent studies point out that spatial synergies exist between biodiversity and other ES [69–70]. Others, however, have documented poor spatial relationships or tradeoffs [40,50]. Still lacking are generalized conclusions linking multiple ES [20,71] and documenting correlations between their priority areas [8,50,72]. This is particularly true for target and non-target ES in watersheds where PHS programs are active.

Forests can help provide a large number of different HS. Such services include climate regulation, water supply, water damage mitigation, provision of water cultural services, among others [21,58]. However, Mexico’s PHS program has focused on the regulation of soil erosion, water quantity and quality. In addition, CONAFOR is also responsible for preparing the national strategy for REDD+ to maintain stored carbon in forests [52,73]. To this end, CONAFOR has launched pilot programs to test payments for carbon credits. However, the launch of a separate national payment program is still several years away. To date there has been no efforts to evaluate how the national PHS program may be influencing levels of carbon storage as non-target ES.

In this paper, we aimed at using spatial analyses to evaluate the targeting performance of Mexico’s national PHS program in central Veracruz. We quantified the effectiveness of existing eligible zones for PHS or properties receiving payments in actually covering areas of high HS provision and social priority, during the first 10 years of program operation (2003–2013). We mapped two target (water yield and soil retention) and one non-target ES (carbon storage). These three services were selected based on their local relevance, the availability of spatial data, and their importance for the PHS program. First, using InVEST we quantified provisioning and described the spatial distributions of ES. Subsequently, pairwise relationships among ES were quantified by using spatial correlation and overlap analyses. Finally, we evaluated targeting performance by: (i) prioritizing areas of individual and overlapping ES; (ii) quantifying spatial co-occurrences of these prioritized areas with those targeted by the PHS; (iii) evaluating the extent to which PHS directly contribute to HS delivery; and finally (iv), testing if these PHS targeted areas disproportionately covered areas with high ecological and social priority. This paper will show that spatial mismatches exist between areas of high ES provisioning and PHS targeted areas.

## Materials and methods

### Study area

**Fig 1:**
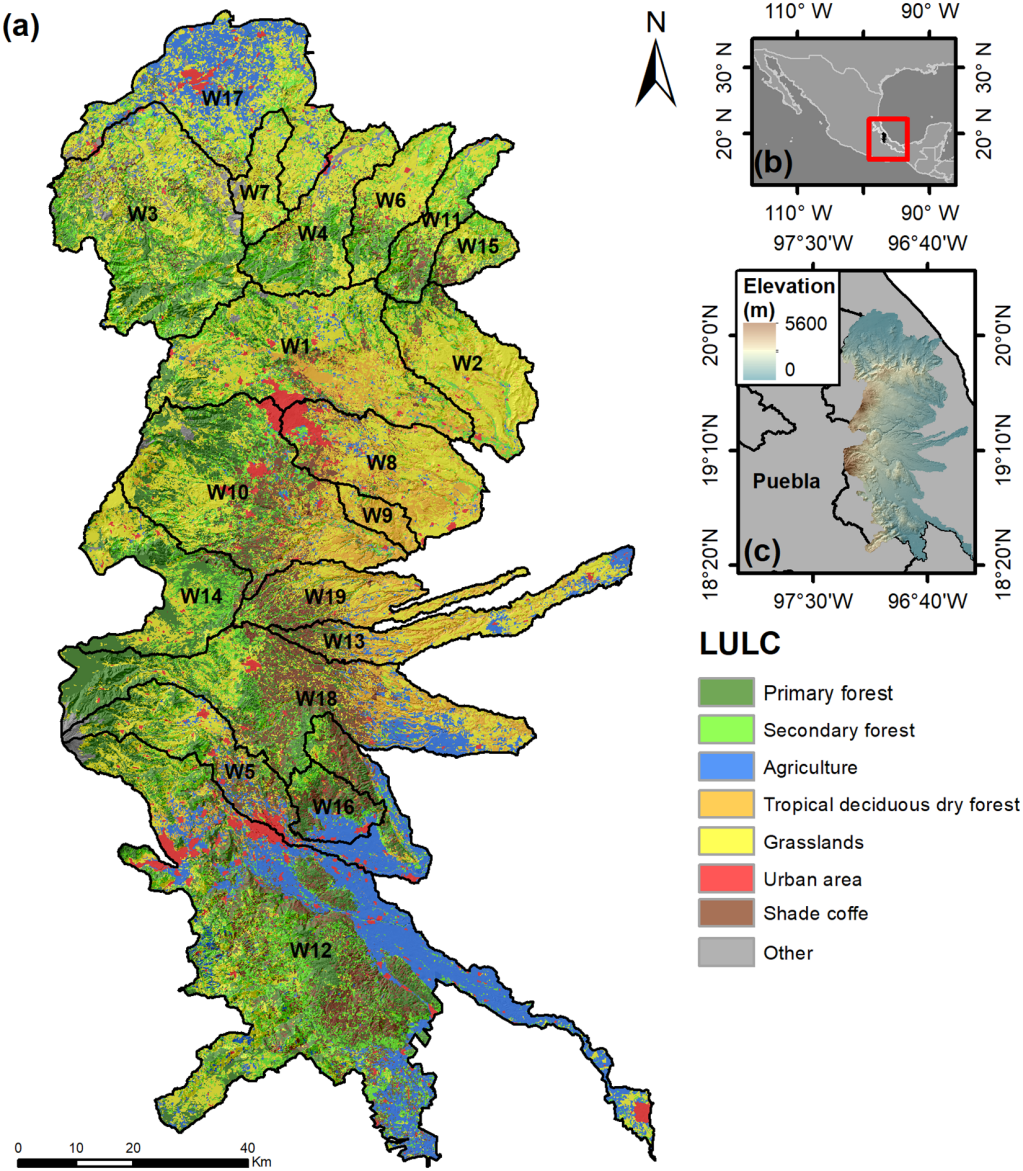
Land-use/land-cover patterns and subwatersheds (19) in the study area (a), located in central Veracruz state, Mexico (b), and the altitudinal variation exhibited (c).

Our study was conducted in subwatersheds in the central highlands of Veracruz state, Mexico (Fig 1a; between 18°34’88”, 20°19’21”N and -96°30’69”, -97°30’27”W). Veracruz is located midway along the Gulf of Mexico (Fig 1b), with rivers channeling 32% of national discharge [74]. Despite its hydrological importance, Veracruz is one of the most deforested states with less than 20% of natural forests remaining [75] and is increasingly affected by tropical storms [76]. This combination of factors has led to declines in HS provisioning, including moderate to severe soil erosion losses and serious declines in water quantity and quality [26]. This has resulted in a considerable threat to well-being of regional communities and in the first experiment with PHS programs in Mexico. Both the national and various local matching-funds programs are currently active in national priority watersheds in the region [74]. The region covers ~9,860 km^2^ running along the slopes of the Sierra Madre Oriental and the Trans-Mexican Volcanic Belt and encompasses 19 subwatersheds. These subwatersheds were selected as spatial units since water-related models in InVEST are run at the watershed scale. Mean altitude ranges from 600–2,300 m, with the highest peak at 5,685 m (Fig 1c). Climate ranges from warm-subhumid to cold-temperate humid in the lower and upper sections, respectively. Mean annual temperature ranges from 17–20°C, while mean annual precipitation varies between 700–3,000 mm/year, with more than 80% occurring during the rainy season (May-October; [77]). Most original forest cover has been replaced by cattle pastures, annual (e.g. sugar cane, corn, etc.), and perennial crops (e.g. shade coffee). Remnant forests are mostly cloud forest and tropical deciduous dry forest in the upper and lower extensions of these subwatersheds, respectively [78–79].

### Land cover map

A land use and cover map (LULC) was derived from 10 multispectral SPOT5 images (<5% of clouds), from April and May 2011 (20m pixel resolution). All images were geo-rectified by precise geometric corrections including image-to-image rectification, using a digital elevation model (DEM) with 20 m intervals. The mean squared error of this correction was less than 1 pixel. A supervised classification was then conducted using the multiresolution segmentation technique and object-oriented classification method in eCognition Developer v.8. A handheld GPS receiver was used to obtain 672 independent ground control and 255 verification points for this procedure. The resulting LULC map (Fig 1a) had a Kappa index of 0.82%, indicating a relatively high degree of interpretation accuracy. Eight major LULC classes were identified, including: primary and secondary temperate montane forests (comprised of cloud, pine-oak, and pine forests), agriculture, tropical deciduous dry forest, grasslands, urban areas, shade coffee, and other (including water bodies, alpine grassland, bare soil, snow, clouds and cloud shadow). We used georeferenced parcels from the National Census of Coffee Producers developed for SAGARPA to help separate shade coffee plantations from forests.

### Data sources and mapping of ES

InVEST is a well-documented tool developed to map, evaluate, and economically value ES to support natural resource use decisions [50]. InVEST has a comparative advantage by allowing users to combine LULC data with information on the supply of ES at different spatial scales [48,80]. Tier 1 models of InVEST v. 3.3 were parameterized using spatial and non-spatial biophysical data from regional studies, literature and national databases. Data from the study region were given priority over more general information sources. Table 1 describes the type and sources of the inputs used in model parameterization, using data for 2013 or as close as possible. Satellite images, the LULC map, and other relevant data layers were processed in ERDAS 8.7 or ArcGis v.10.2. Tier 1 models of surface water yield, soil retention and carbon storage were used to quantify and map spatial distributions of ES, at a spatial resolution of 20 m [81–82]. Below, we provide a brief description of each model. Detailed model description and their accuracy in assessing ES provision are provided in the supporting information (S1 File). These models are also described in Tallis et al. [81] and Kereiva et al. [83].

**Table 1:** Data sets used for mapping ecosystem services in InVEST. All models used the LULC and subwatershed maps described in the text.

*Model*	*Data type*	*Unit*	*Source and description*
***Carbon storage***			
Aboveground	*Table*	*ton ha*^*-1*^	National forest and soil inventory (2011) and IPCC (2006)*
Belowground	*Table*	*ton ha*^*-1*^	National forest and soil inventory (2011) and IPCC (2006)*
Dead matter	*Table*	*ton ha*^*-1*^	National forest and soil inventory (2011) and IPCC (2006)*
Soil organic matter	*Table*	*ton ha*^*-1*^	National forest and soil inventory (2011) and IPCC (2006)*
***Surface water yield***			
Digital elevation model	*Raster (15m)*	*m*	*INEGI (2012)*. www.inegi.org.mx *
Annual precipitation	*Raster(20m)*	*mm y*^*-1*^	*Hydrological database ERIC III*. *(IMTA*, *2006)* www.conabio.gob.mx*
Potential evapotranspiration	*Raster(20m)*	*mm y*^*-1*^	*Estimate from Hydrological data base ERIC III (IMTA*, 2006)*
Maximum soil depth	*Raster(20m)*	*mm*	*Estimate from national database of soil profiles*. www.inegi.org.mx*
Plant available water content	*Raster(20m)*	*0–1*	*Estimate from national database of soil profiles*. www.inegi.org.mx *(Saxton*, 1996)*
Root depth	*Table*	*mm*	*Tallis et al*. *(2010) and FAO Irrigation and Frainage (Droogers and Allen*, 2002)*
Evapotranspiration coefficients	*Table*	*0–1*	*Tallis et al*. *(2010) and FAO Irrigation and Frainage (Droogers and Allen*, 2002)*
***Soil Retention***			
Soil erodibility (k factor)	*Raster(20m)*	*MJ mm ha y*^*-1*^	*Estimate from national database of soil profiles (Torri et al*., 1997)*
C and P factor	*Table*	*Dimensionless*	*Tallis et al*. *(2010)*
Rainfall erosivity (R factor)	*Raster(20m)*	*ton h MJ-1 mm*^*-1*^	*Estimate from database ERIC III*. www.conabio.gob.mx *(Roose*, 1996)*
Sediment retention coefficients	*Table*	*ton ha*^*-1*^ *y*^*-1*^	*Renard et al*. (1991)*
*Subwatersheds*	*Vector*	*Dimensionless*	Shared Risk Trusteeship (FIRCO-SAGARPA 2006)*

* References are cited in the supplementary information section

Surface water yield: This model is based on an approximation of the Budyko curve [84–85] and annual average precipitation. Annual water yield (mm y^-1^; WY) is defined as the amount of water runoff across the landscape [81]. Total water yield is estimated (per pixel) as the contribution from each pixel of the landscape, considering how specific characteristics of LULC types affects runoff and evapotranspiration, and then subtracting these from the average annual precipitation [83,85]. The model simplifies water movement by combining the flow of groundwater and surface water, under the assumption that groundwater follows the same flow path as surface water and eventually reaches a stream, where it is discharged [86]. Values per pixel are the summed to provide a total yield for the watersheds.

Soil retention: This model estimates the ability of different LULC to retain soils and prevent erosion [50]. It first computes annual potential soil loss or sediment loads for each pixel of the landscape (ton ha^-1^ yr^-1^), using the Universal Soil Loss Equation (USLE; [81,87]). The model then routes soil loads originating from each pixel along its flow path, with vegetated pixels retaining a percentage of such losses from upstream pixels, depending on the ability of present vegetation to slow erosion, and then exporting the remainder downstream [13,88]. Soil retention is computed by calculating the difference between potential soil loss and percentage of erosion retained by vegetation.

Carbon storage: This model is a simplification of the carbon cycle and generates a map of total carbon (Mg C ha^-1^). The resulting output represents the sum of estimated stored carbon values from aboveground and belowground biomass, organic matter, and dead carbon (litter combined with other dead organic matter). This information is obtained via coefficient tables for each LULC type [81,88]. We parameterized the different carbon pools based on local information and national data (see S1 File).

Model outputs were verified by comparing the resulting estimates (annual average water yield and soil retention) of three main subwatersheds to local observed data of CONAGUA at interest points. While not all LULC types are eligible to receive PHS, they were included in models to quantify ES provision and identify spatial patterns over the entire study region. InVEST models estimate HS provision at the watershed scale but are also configured for operating at smaller hydrologic response unit or quantify provisioning at pixel scale. The later scale was used as spatial units in order to analyze spatial heterogeneity in each type of ES distribution.

### Analysis of spatial relationships

We generated maps of priority areas for individual ES (WY: water yield; SR: soil retention; CS: carbon storage), combined hydrological services (HS: WY+SR), and for multiple ES (MS: HS+CS). Since models estimated ES provisioning in specific units, we standardized the raster grid values using a common scale ranging from 0 to 1 (low to high provision levels). We followed the methodology proposed by Ferrano [64] and Lavorel et al. [67]. Then we subtracted from each pixel the minimum value of each ES map and dividing the difference by the range of each ES [70]. To spatialize the upper range of ES provision, percentiles of the resulting frequency distributions were then computed to classify pixels with different degrees of importance and to reach 20% of the total area for each ES [8,10,89]. Priority areas (PAs) were set as the upper quintile (80^th^ percentile and above), whereas medium and low priority were those in the third and fourth, and first and second quintiles, respectively. This cutoff was used to ensure comparability among priority areas, where each ES had approximatively the same capacity to be provided. Similar ranges were used in previous studies [35,46,68], where a cutoff of the upper 10% or less of pixels delimited their priority areas.

We quantified the direction and strength of the spatial relationships (trade-offs or synergies) between ES in two steps. First, we performed Pearson’s correlation analyses to assess pairwise relationships among ES, using a subset of overlapping pixels for each ES map (n = 10,500) [90]. Standardized maps for each ES were used to give more uniform distribution to the analysis [36]. We used SAM v4.0 (Spatial Analysis in Macroecology) and R v.2.15.2 (http://www.r-project.org/) for statistical analyses. Relationships with a Person’s correlation coefficient over 0.3 were deemed strong and whereas those between 0.1–0.3 were deemed weak correlations [90–91]. Subsequently, we compared spatial configuration and pattern (location, patchiness and spatial aggregation) of all three maps. We used Global Moran’s I analysis in SAM to identify and determine average distance of pixels grouping according to their provision levels.

To identify and map spatial ES co-occurrences, individual priority maps (top 20% of streamside pixels) were weighted and summed. We analyzed the spatial distribution of priority areas for overlapping HS (where priority areas for water yield and soil retention overlapped) and multiple overlapping ES (where all priority areas overlapped) [19]. We used the Fuzzy Set theory [24,85] and the Fuzzy Overlay Analysis tool in the Spatial Analysis extension of ArcMap.

### Overlap between PHS target areas and priority areas

We evaluated the capacity of existing areas targeted by PHS to cover areas of both ecological and social priority. Shape files for eligible zones (EZs) and properties actually receiving PHS (hereafter denominated “payment zones”; PRs) were obtained from CONAFOR (2015) for the period 2003–2013. Since these areas changed slightly each year, data layers for all years were combined into a single coverage for each type of zone. Marginalization index was used as our measure of social priority. To generate the social priority map we used the ordinary Kriging interpolation methods in ArcGIS 10.2., to transform the aggregated marginalization index into a 20 m raster (ranging from -1.56 to 2.07). This index was obtained from CONAPO [92] for each locality of the study region and results from the aggregation of eight socioeconomic indicators (e.g. education, quality of water and sanitation system, land ownership and availability of electricity). High social priority was determined using the threshold proposed by CONAPO for the condition of “very high marginalization” (<0.712).

Spatial targeting effectiveness of eligible and payment zones in covering prioritized areas for individual or overlapping ES was assessed in two steps. First, we computed the proportion of areas within each subwatershed (n = 19) covered by each priority area. Next, overlap analyses were performed with eligible and payment zones to calculate the proportion of each priority area occurring within them [24,50,93]. For these analyses, we used null hypotheses of independent spatial distributions between priority areas and zones targeted by PHS. We based on the assumption that both simple proportions of priority areas found within the PHS zones in each subwatershed belong to the same underlying distribution. We used T-tests to compare the proportion of priority areas observed within eligible zones vs. expected proportions derived from actual coverages in each subwatershed. We then repeated this test using observed proportions within payment zones vs. the expected proportions within associated eligible zones. This second analysis was necessary since payments are not allowed outside of eligible zones. Regression analyses and analysis of variance (F-tests; 95%) were used to determine whether the slopes of the regression lines for expected and observed values were significantly different.

We then assessed PHS capacity to cover areas for both high priority and high marginalization index. We used overlap analyses to identify spatial co-occurrences and potential priority areas with overlapping HS and social priority. We repeated this to identify priority areas with overlapping ES (all three services) and social priority. To evaluate PHS effectiveness in targeting both goals, we overlapped such prioritized areas with zones targeted by PHS and followed the procedure described previously to identify spatial co-occurrences. Finally, an additional randomization test was performed to assess whether the choice of the upper 20th or 33th percentile as a cutoff for high priority would have resulted in greater overlap to asses robustness of previous estimations [13,68]. All analyses and interpretations were carried out using R v.2.15.2. Finally, we evaluated the extent the existing PHS targeted areas provide ES. Then, we over-laid these areas on each of the priority area maps and calculated statistics to provide a measure of the amount of ES that they supposed to deliver.

## Results

### Spatial patterns of service provision

**Fig 2:**
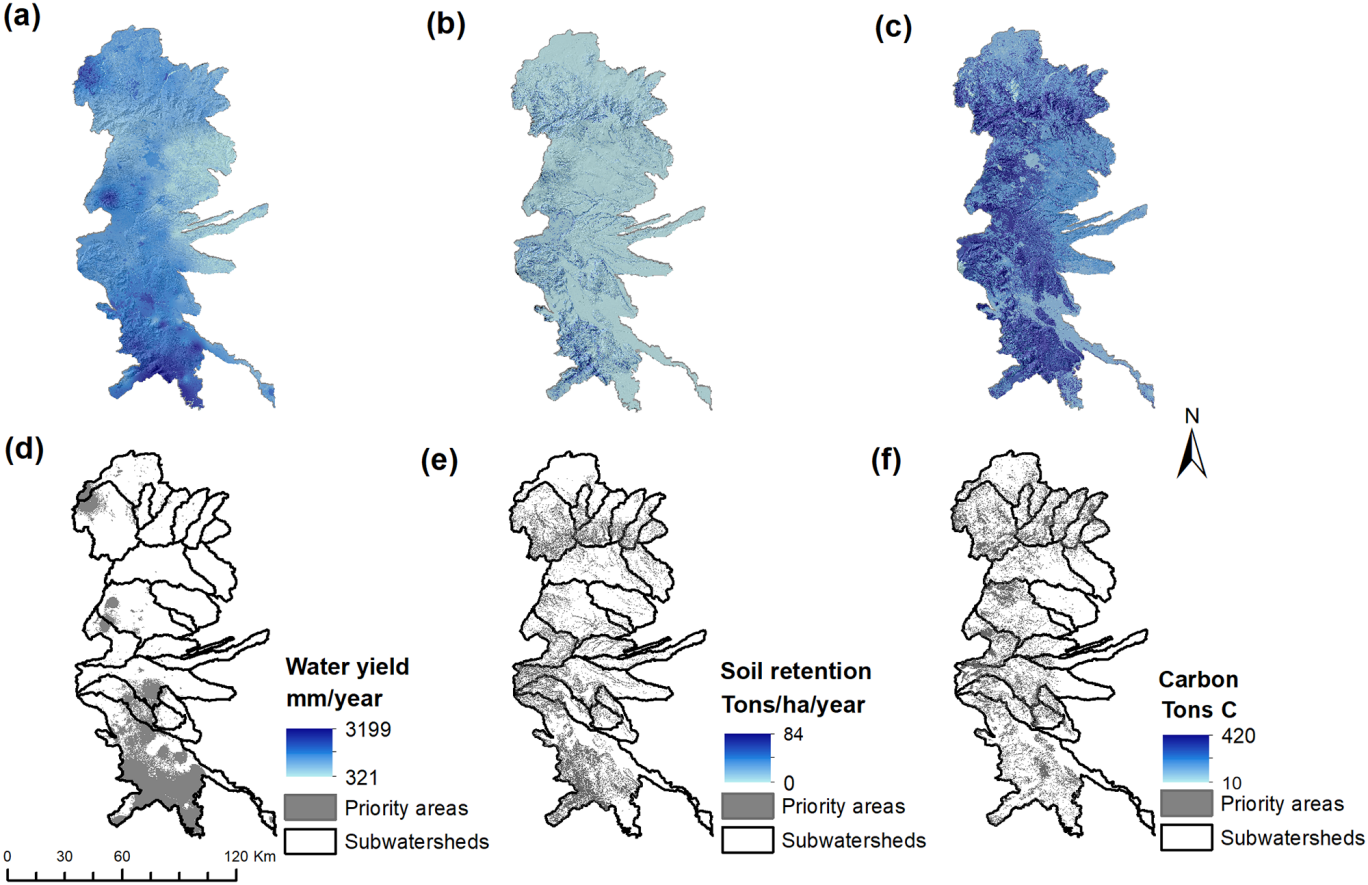
Modeled spatial distributions of ecosystem service provision (a-c); and associated priority areas (top 20% of pixels; d-f).

Provision levels and spatial distributions of predicted priority areas for individual ES are illustrated in Fig 2. Factors influencing provisioning for each ES varied considerably, resulting in a spatially heterogeneous distribution. Most important areas for water yield were located in the mid-to upper-elevations of the central and southern portions of the Sierra Madre mountains, where the highest amounts of rainfall typically occurs (Fig 2a and S1 Fig). While variations between the distribution of water yield and soil retention was expected to be relatively low, contrasting spatial patterns were observed (see Fig 2a and 2b). This contrast was most likely due to the spatial distribution of geomorphological conditions (distribution of soil erodibility and erosivity factors, as well as slopes) and riparian corridors important in modelling soil and sediment dynamics. As expected important areas for carbon storage were more closely associated with forested areas, particularly those located in upper elevations (Fig 2c).

Since HS delivery depends not only on LULC types, but also on meteorological factors, topography, and soil characteristics (See S1 and S2 Figs), we observed a wide range of levels of provisioning within each LULC type. Prioritized areas for mean annual water yield were spatially associated with a diversity of land covers, particularly secondary forest, shade coffee, and agricultural lands (as shown in Table 2). However, water yield did not differ considerably between these land uses. In contrast, of the priority areas for soil retention services were mostly associated with primary (32%) and secondary forests (24%). As LULC is the determinate factor in carbon calculations, predicted priority areas were overwhelmingly associated with primary forest and secondary forest as expected. While minimal differences were observed in the mean values of soil retention for primary and secondary forests, there was a notable variation in estimated carbon values for the same land covers. For HS, these results suggest that estimations of service delivery may not be based on the conservation of standing forest alone.

**Table 2:** Pearson’s correlation analysis between pairwise ES and clustering Moran I index.

	Water yield	Soil retention	Carbon storage	Moran’s I	Significance
**Water yield**	1			0.99	P<0.001
**Soil Retention**	0.35	1		0.91	P<0.001
**Carbon storage**	0.12	0.47	1	0.86	P<0.001

Note: High correlation (dark gray; >0.3), weak correlation (light gray; 0.1–0.3).

Moran’s I analyses showed that the spatial distribution of predicted priority areas for individual ES differ significantly in the region (as shown in Table 3). Prioritized areas for soil retention and carbon storage were found significantly and spatially clustered (auto-correlated). However, such areas were scattered throughout the region in several smaller patches. The spatial configuration of priority areas for water yield exhibited the highest spatial clustering in larger more aggregated patches. Pearson correlation among pairs of ES showed that clear synergies exist among ES. We observed that a strong positive correlation exist between water yield and soil retention (*p* < 0.01). However, strongest still is the relationship between soil retention and carbon storage services (*p* < 0.01). This relationship is probably due to woody land cover being necessary for achieving high provision levels for these services. Prioritized areas for soil retention are probably also related to topography, since steeper areas favor conservation of natural forest remnants (see Figs 1 and 2, and Table 3). Spatial correlations between prioritized areas for carbon storage and water yield were significant but weaker than other pairwise comparisons. Areas important for water yield were clustered in areas adjacent to shade coffee and agriculture areas rather than riparian corridors and locations in the upper subwatersheds where carbon storage was potentially high.

**Table 3:** Overlap between ES priority areas and principal land use/land cover types, and their range of ES provisioning.

Units	Provision range	Principal land uses	%	Average
**Water yield** mm yr^-1^	1,570–3,199	Secondary forest	23	1,938 ± 275.2
		Shade coffee	19	1,902 ± 277.7
		Agriculturet	17	1,923 ± 270.7
		Primary forest	16	1,842 ± 219.1
		Grassland	11	1,830 ± 203.3
**Soil Retention** ton ha^-1^ yr-^1^	32–84	Secondary forest	35	74.9 ± 5.2
		Primary forest	24	69.8 ± 7.8
		Grassland	21	62.9 ± 9.3
		Shade coffee	12	74.3 ± 12
		Topical deciduous forest	5	47.4 ± 6.4
**Carbon storage** ha^-1^ yr^-1^	310–420	Primary forest	71	417 ± 28.3
		Secondary forest	16	312 ± 26.5
		Shade coffee	6	140 ± 62.5
		Grasslands	5	121 ± 37.5
		Agriculture	1	100 ± 57.2

**Fig 3:**
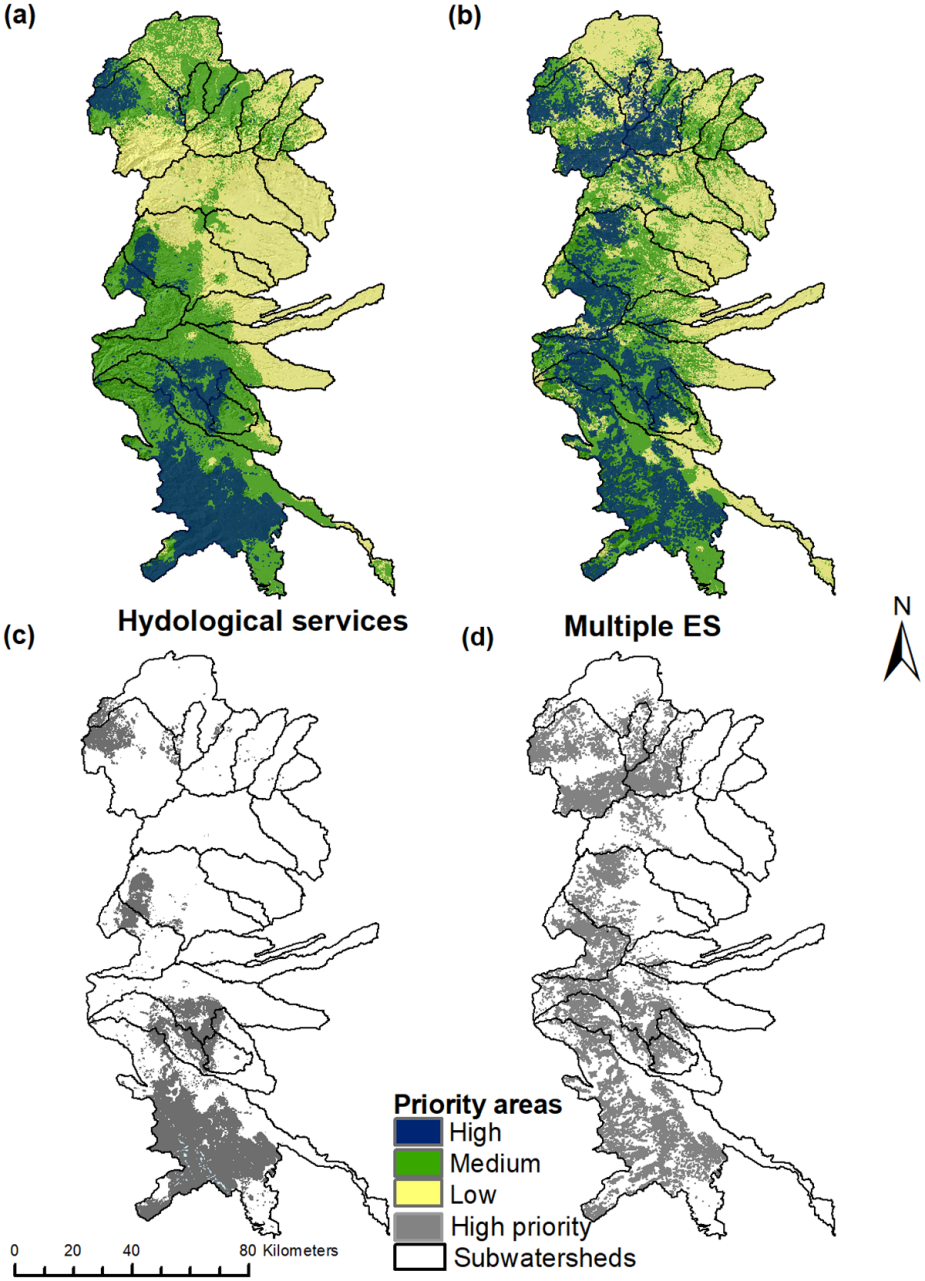
Modeled provision of hydrological and multiple ecosystem services (a,b), and associated priority areas (c,d).

Fig 3 shows the spatial overlaps of priority areas for combined hydrological services and multiple ES (combined HS + carbon storage). Overlap analyses revealed that approximately 5.5% of the study region (~54,451 ha) was classified as high priority for HS (Fig 3a). These areas represent the spatial co-occurrence of the top two quintiles for soil retention and water yield services. Areas that scored the highest for all three services and co-occurred, were subsequently identified as priority areas for multiple ES and were somewhat rare than priority areas for HS, representing only 4.7% of the region (~44,897 ha; Fig 3b). While there are few areas providing high levels of all ES, prioritized areas for more than one service were relatively large (see Fig 3) and could be considered in efforts to bundle ES. Our results also highlight the contrasting distributions and possible trade-offs between ES and among the two HS considered here, and that could complicate the goal of conserving target and non-target ES through PHS.

### Spatial overlap between targeted zones for PHS and ES

**Fig 4:**
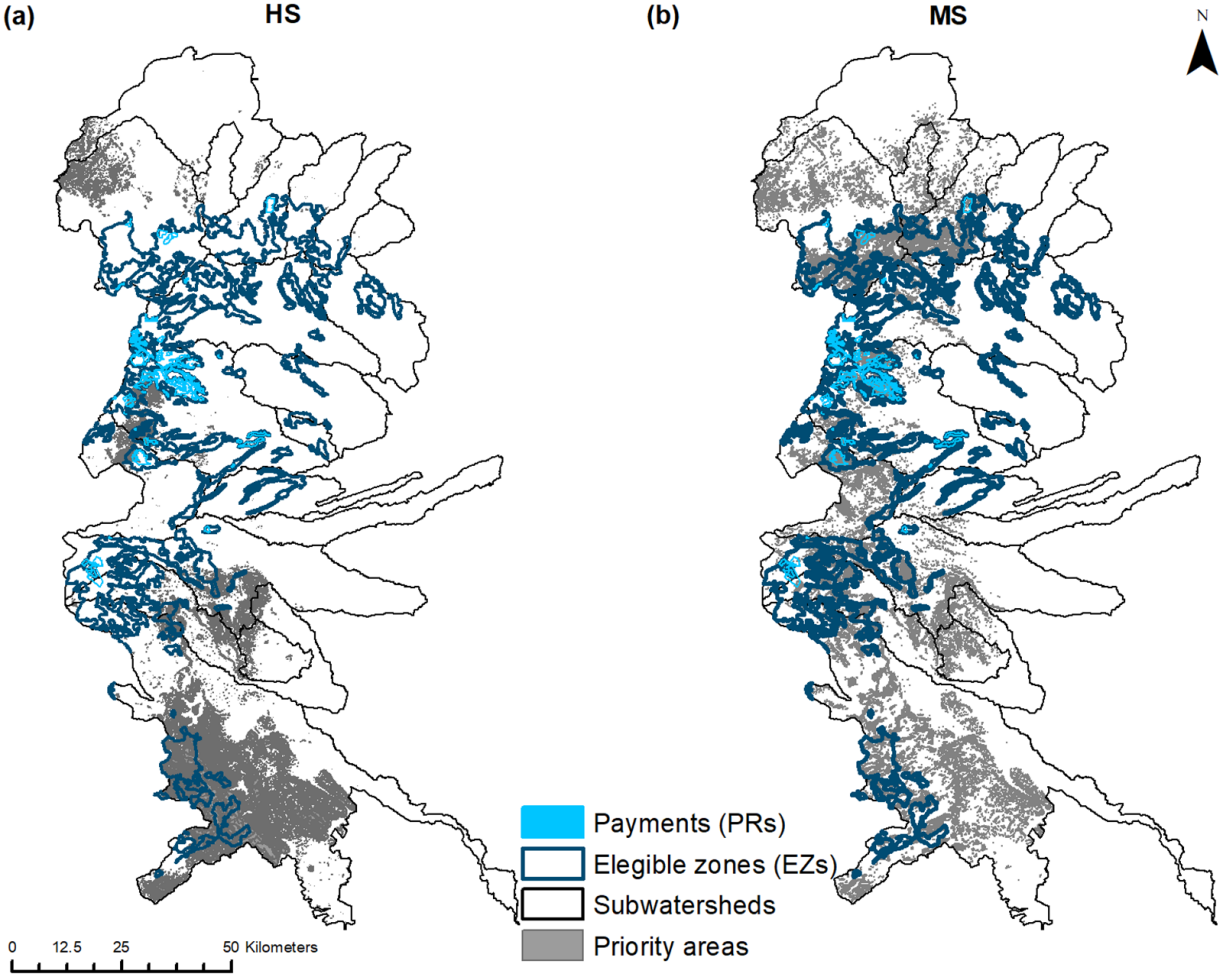
Spatial overlap between priority areas for hydrological (a) and multiple ES (b) with eligible (dark blue) and payment zones (light blue).

The spatial overlap between PHS zones and priority areas for individual and combined ES showed similar heterogeneity. Eligible zones covered a total of 14% of the study region (~138,243 ha), and varied considerably among subwatersheds (Fig 4). These zones were more concentrated in mid-to-upper altitudes, particularly in central and southern subwatersheds. However, spatial overlap analysis show that modelled priority areas for HS and multiple ES were generally not well covered by these zones. The highest spatial overlaps identified were between eligible zones and priority areas for carbon storage (38%), followed by water yield, and areas of multiple overlapping ES (Table 4). Payment zones occupied only 11.3% of the zones that were eligible for PHS (1,577 properties covering ~15,655 ha), or 1.6% overall within the subwatersheds. Payment zones were concentrated in the central portion of the region (Fig 4). However, exhibited markedly lower spatial overlap with priority areas for individual and overlapping ES than that observed for the eligible zones. Only ~11,660 (8.4%) and ~291 (0.5%) ha of eligible and payment zones were found to be important in covering prioritized areas for combined HS, respectively (Table 4).

**Table 4:** Overlap between modeled priority areas for ES and PHS targeted zones.

*Percentages of proportional overlaps*					
	Water yield	Soil retention	Carbon storage	HS	MS
**Eligible zones**	13.8	4.4	37.9	8.4	11.9
**Payment zones**	1.4	0.2	5.0	0.5	13.8

Note: hydrological services (HS), and multiple ES (MS)

Analyses revealed considerable inter-watershed variation in the distribution of predicted priority areas and zones targeted by PHS, including cases where such areas were completely absent in certain subwatersheds (Table 5). Variability in the coverage (%) of priority areas for individual ES within subwatersheds was most pronounced for water yield, followed by carbon storage, and soil retention services. Inter-watershed variation for combined HS and multiple ES were comparatively lower (Table 5A). Variation in the coverage of subwatersheds by eligible zones was substantially higher than that for payment zones, since the latter were absent in an important number of subwatersheds even when eligible zones were present. Overlap analysis indicates that there was a considerable decline in coverage of priority areas downscaling from the whole subwatershed to eligible zones (Table 5B). For example, comparing averages at these disparate scales reported in Table 5A and 5B, there was a 10-fold reduction in coverage of priority areas for water yield (9.3 vs. 0.9%). Similar, but even more pronounced differences were noted for comparisons at the scale of payment zones with the exception of carbon storage, whose coverage increase slightly. Overall, there was a trend of these eligible and payment zones underrepresenting important areas for individual or combined HS at both spatial scales. Which revealed that PHS targeted zones are poor design in terms of its location and may need to be modified in order to maximize the provisioning of evaluated HS, and other services such carbon storage.

**Table 5:** Variation between subwatersheds in the areas occupied by PAs, EZs, and PRs (A), and percentage of overlap of PAs found within EZs and PRs within EZs in each subwatershed (B).

A									B				
	*Percentages of the subwatersheds covered by all PAs and PHS targeted areas*								*Proportional overlaps between PAs with EZs (or within PRs)*				
*Sub-WS*	*Area* *(ha)*	*WY* *%*	*SR* *%*	*CS* *%*	*HS* *%*	*MS* *%*	*EZs* *%*	*PRs* *%*	*WY* *%*	*SR* *%*	*CS* *%*	*HS* *%*	*MS* *%*
**W1**	79690	0.1	8.5	11.8	0.0	1.2	22.8	0.90	0.0 (0.0)	0.2 (0.0)	7.0 (1.2)	--	0.6 (0.0)
**W2**	38253	0.0	11.8	5.6	0.0	0.0	12.9	0.0	--	0.2	4.5	--	--
**W3**	86339	15.3	29.9	34.3	1.7	10.5	22.5	1.55	0.1 (1.1)	1.3 (0.3)	16.6 (5.3)	0.01 (0.0)	5.4 (1.1)
**W4**	34018	2.0	28.2	31.1	0.0	12.4	31.7	1.27	0.0 (0.4)	2.1 (0.2)	26.9 (3.9)	--	10.1 (0.4)
**W5**	58268	26.9	22.0	21.7	3.9	5.5	14.2	0.45	1.3 (0.1)	0.7 (0.4)	6.7 (1.1)	0.7 (0.0)	2.2 (0.1)
**W6**	24143	1.1	17.5	29.2	0.0	0.3	22.9	0.0	0.1	1.1	15.8	--	0.2
**W7**	13578	5.9	22.8	22.1	0.2	5.5	3.5	0.0	0.0	0.2	3.3	0.0	0.9
**W8**	45037	0.0	3.8	1.5	0.0	0.0	5.6	0.0	--	0.0	0.8	--	--
**W9**	7255	0.0	5.7	1.0	0.0	0.0	16.4	0.0	--	0.1	2.1	--	--
**W10**	84500	9.5	12.8	23.6	2.1	2.8	24.5	10.28	3.2 (3.2)	0.4 (0.8)	12.9 (25.9)	1.1 (1.0)	1.9 (3.2)
**W11**	21100	0.2	24.1	34.1	0.0	0.0	10.2	0.0	0.0	0.5	7.9	--	--
**W12**	208656	62.5	30.9	19.2	21.0	7.4	10.1	0.69	6.4 (0.0)	0.9 (0.2)	4.7 (4.4)	4.5 (0.0)	1.2 (0.0)
**W13**	29714	0.6	5.4	5.4	0.0	0.2	0.0	0.0	0.0	0.0	0.0	--	0.0
**W14**	39596	10.5	28.5	37.0	2.1	8.8	13.5	3.00	2.6 (5.8)	0.5 (0.7)	9.0 (20.8)	0.1 (1.6)	2.4 (5.6)
**W15**	11411	0.3	22.1	20.0	0.0	0.0	0.7	0.0	0.0	0.0	0.6	--	--
**W16**	14850	14.4	17.8	30.0	1.6	6.1	0.0	0.0	0.0	0.0	0.0	0.0	0.0
**W17**	68587	10.5	3.8	14.6	0.4	0.8	0.0	0.0	0.0	0.0	0.0	0.0	0.0
**W18**	90579	15.4	21.6	20.9	3.7	4.6	16.1	1.75	1.3 (0.03)	0.8 (0.2)	9.4 (5.3)	0.7 (0.0)	1.7 (0.03)
**W19**	29532	0.9	14.9	9.8	0.0	0.3	10.1	0.0	0.0	0.2	2.2	--	0.0
**Average**		9.3	17.5	19.6	1.9	3.5	12.5	1.0	0.9 (1.3)	0.5 (0.4)	6.9 (8.5)	0.8 (0.4)	1.9 (1.3)
**Variance**		223.8	83.5	130.2	23.1	16.2	89.6	5.7	3.2 (4.4)	0.3 (0.1)	50.5 (88.6)	2.1 (0.5)	7.7 (4.4)
**SD**		15.0	9.1	11.4	4.8	4.0	9.5	2.4	1.8 (2.1)	0.6 (0.3)	7.1 (9.4)	1.5 (0.7)	2.8 (2.1)
**Min**		0.0	3.8	1.0	0.0	0	0.7	0.0	0.0 (0.0)	0.0 (0.0)	0.0 (1.1)	0.0 (0.0)	0.0 (0.0)
**Max**		62.5	30.9	37.0	21.0	12.4	31.7	10.3	6.4 (5.8)	2.1 (0.8)	26.9 (25.9)	4.5 (1.6)	10.1 (5.8)

Note: Water yield (WY), soil retention (SR), carbon storage (CS), hydrological services (HS), multiple ES (MS), eligible zones (EZs), and properties receiving payments (PRs)

Formal tests (Student *T*) to determine whether proportional coverage of priority areas with PHS targeted zones (as reported in Tables 4 and 5) were over or under represented compared to expected levels from random placement are reported in Table 6. Comparisons between the level of coverage of priority areas at the subwatershed scale (expected values) to those found in eligible zones (observed values) revealed significantly lower than expected overlaps for all individual and overlapping ES (n = 19; Table 6A). A similar but less consistent pattern was detected in comparisons of the coverage of priority areas within eligible zones (expected values) vs. those found within payment zones (observed values). These spatial overlaps were significantly lower than expected coverages, except for water yield and overlapping HS, where coverage was also low but did not differ from that expected by random placement (n = 8; Table 6B).

**Table 6:** Coefficients from the relationships between expected and observed proportional overlap values.

*Subwatersheds vs*. *EZs*						
**A**	***T-test***			***F-test***		
	*t*	*p-value*	*Mean difference*	*F*	*p-value*	*Variances*
**WY**	-2.311	0.035*	-8.474	0.012	0.000*	0.012
**SR**	-8.873	0.000*	-18.506	0.004	0.000*	0.004
**HS**	-3.747	0.001*	-2.097	0.047	0.000*	0.047
**CS**	-6.009	0.000*	13.158	0.389	0.057	0.371
**MS**	-5.169	0.000*	-10.946	0.181	0.002*	0.181
**B**						
*EZs vs*. *PRs*						
**WY**	-2.345	0.051	-15.711	0.021	0.000*	1.599
**SR**	-7.637	0.000*	-22.446	0.001	0.000*	3.596
**HS**	-1.518	0.051	-7.944	0.021	0.000*	1.876
**CS**	-4.725	0.002*	-16.465	1.236	0.007*	1.091
**MS**	-6.565	0.000*	-11.093	1.227	0.007*	3.264

*P<0.05.

Note: Water yield (WY), soil retention (SR), carbon storage (CS), hydrological services (HS), multiple ES (MS)

**Fig 5:**
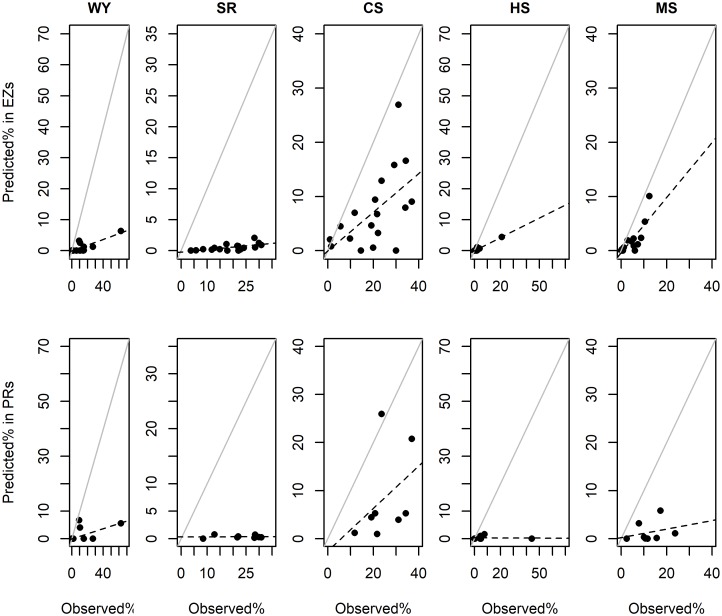
Relationship between observed and expected (predicted) proportional overlap values, for all priority areas with zones eligible within subwatersheds (EZs; *n* = 16) and payment zones in these EZs (PRs; *n* = 8). Note that the x-axis is observed values (dotted lines), while the y-axis is predicted value (solid lines).

Spatial mismatches were detected between zones targeted for PHS and prioritized areas for ES supply, clearly illustrated in Fig 5. Relationships between observed and expected overlap values were lower than expected. Analyses using the hypothesis of equality between regression lines (*F-test*; Table 6) revealed that slopes were significantly lower than expected 1:1 lines. This pattern held for all individual and overlapping ES evaluated at both spatial scales of comparison (Table 6 and Fig 5). The sole exception to this trend were the priority areas for carbon storage found within the eligible zones at the subwatershed scale, where no significant difference between regression lines was observed. Fig 5 represents the distance from predicted (expected) proportional overlaps to the 1:1 line from observed overlaps, and the position of points relative to these lines indicates which ES were best targeted. Thus, while carbon storage had the highest spatial overlap overall with eligible zones than other ES, subwatersheds still uniformly fell below this line and thus showed a similar trend. In general, our results showed that the targeting effectiveness of existing eligible and payment zones in covering priority areas for the ES we studied, was no better that random (and frequently worse) in our study region. We repeated our initial analyses using the top 33% of pixels (instead of 20%) for identifying priority areas for individual and overlapping ES. However, none of our results produced any significant deviations from these patterns and suggest our findings were robust.

### Capacity of PHS in achieving ecological and social targets

**Fig 6:**
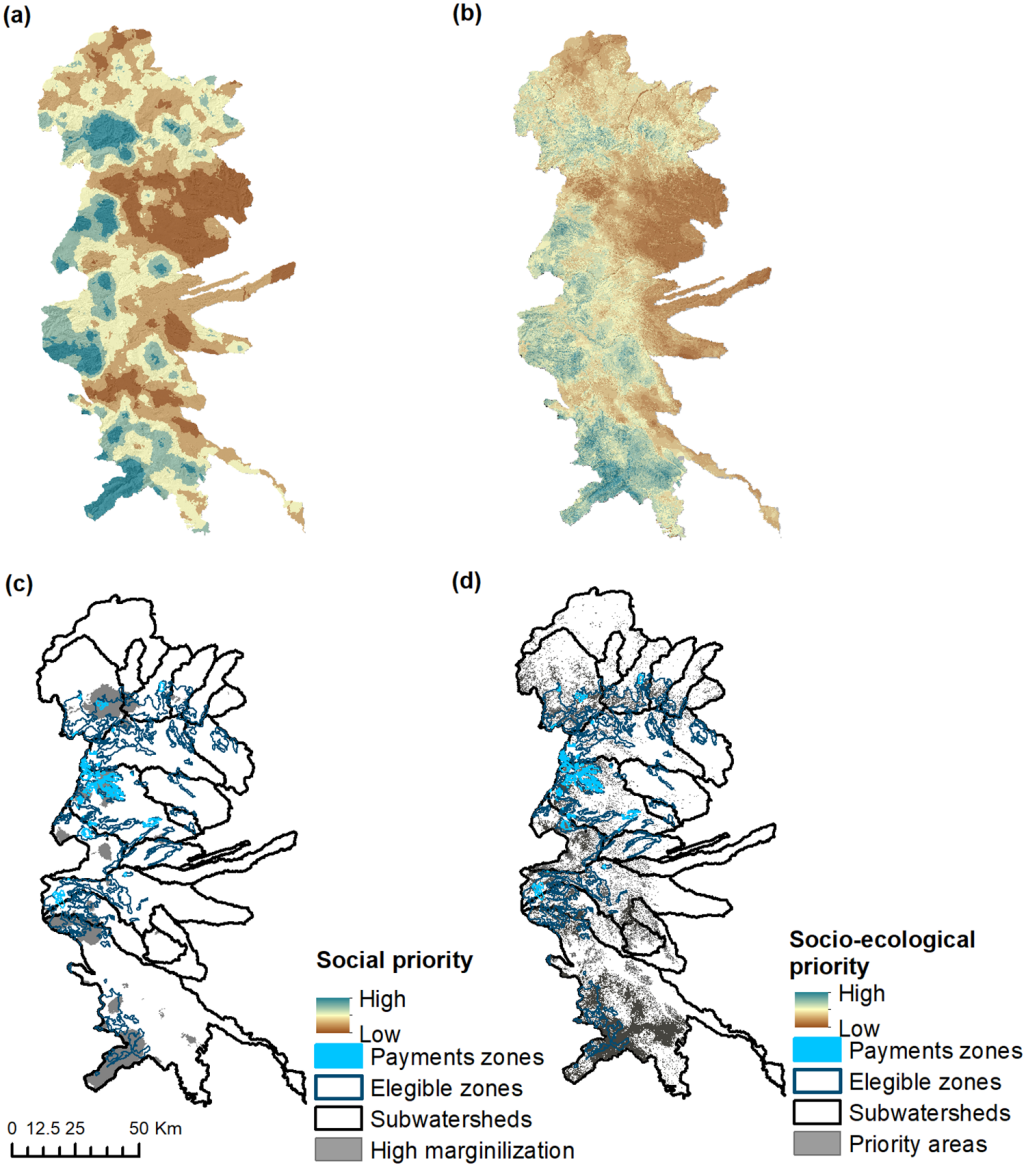
Distribution of interpolated marginalization index (a); prioritized areas of high ecological and social priority (b). Also are shown areas for high social priority (c) and the overlap between areas with hydrological and social priority with the zones targeted by PHS.

The interpolated marginalization map we generated to assess whether PHS simultaneously cover social priority areas, while also covering priority areas for HS, exhibited values ranging between -1.5 and 2.0 (Fig 6a). Using our selection threshold of 0.71 (high marginalization) for high social priority yielded an area of 88,195 ha, of which 39% and 4% spatially overlapped with eligible and payment zones, respectively (Fig 6b). By combining these maps with priority areas for HS (Fig 3a) we identified potential areas for maximizing both ecological and social goals (as shown in Fig 6c). Approximately 10.5% of the study region, amounting to 101,913 ha, were areas that ranked at the top quintiles for both sets of criteria (Fig 6d). Fig 6 shows that, as expected, bigger differences in the spatial distribution occurred between eligible and payment zones, and these for under both goals. About ~11,000 ha were covered by eligible zones and a total area of around ~1,338 ha were found within payment zones. Most areas were located in the southern part of the study area and several smaller patches were found in the central and northern regions.

Finally, Table 7 shows the gap in effectiveness of current eligible and payment zones. It should be also noted that actual performance of eligible and payment zones shows how inefficiently such areas provides services compared with all priority areas. Overall, with eligible zones providing only 9.5% (901.13 M m^3^ yr^-1^) and 1.3% (122.40 M ton ha^-1^ yr^-1^) of total production of water and retained soils by their respective priority areas, respectively (Table 7A). Eligible zones performed well in providing 25% of the total amount of carbon stored in the different carbon pools by the priority areas. Table 7A, represent the provisioning for each ES by their priority areas and the amount reached by the zones targeted by PHS, and those with high social and ecological priority that overlapped with these priority zones. As shown in Table 7B, there was high variation in estimated ES between the zones targeted by PHS, priority areas, and these for ecological and social goals. For example, provisioning of water yield per ha was higher (42,424 m^3^ ha^-1^ yr^-1^) that these observed values for the PHS targeted zones, followed by the areas with both ecological and social priority. The amount of retained soils varied little between these areas. In general, a lower level of ES provisioning per unit area (ha) was observed for the zones targeted by PHS. Considering modeled priority areas as possible targeted areas allows for more efficient provisioning of the ES we examined.

**Table 7:** Comparison of the effectiveness of eligible and payment zones found in each priority area vs. respective priority areas (A) and levels of provisioning per unit area (ha).

	A				B			
	Net production of ES within priority areas				Provision level per ha			
ES	Eligible zones	Payment zones	Priority areas	Socio-ecological	Eligible zones	Payment zones	Priority areas	Socio-ecological
**WY** mm yr^-1^	901.13	122.40	9387.40	2,830	29,264	29,664	47,637	42,424
**S**R ton ha^-1^ yr^-1^	106.4	7.38	332.27	204.95	72.3	63.6	79.8	75.7
**CS** ha^-1^ yr^-1^	19.74	3.10	77.43	20.27	288	316	394	337.3

Note: values are given in millions of tons of stored carbon, tons per year of retained soil, and m^3^ per year of water yield (A). Also are given values in ES metrics per hectare (B), for water yield (WY), soil retention (SR), carbon storage (CS).

## Discussion

The quantitative analysis reported in this study suggest that the targeting effectiveness of Mexico’s national PHS program, during it’s first 10 year of operation (2003–2013), could be substantially improved through the spatial prioritization of HS. According to Muñoz-Piña et al. [28,53], this program has sought to target critical forests that were assumed to insure HS provisioning, while also supporting poverty alleviation. However, our findings contrast with previous evaluations of program effectiveness in Mexico. These studies used criteria such as avoided deforestation to evaluate how the absence or presence of payments affect factors such as additionality [27,30–32], and leakage (e.g. ES losses from areas where PHS are located) [94–95]. Studies performed nationally highlighted impacts of PHS on deforestation that appears to be positive but low and attribute this finding to the programs weak capacity to channel funding to the most threatened forests [28,54]. In contrast, analyses performed at regional scales found that PHS succeeded in enrolling forests with high deforestation risk [30,32,53]. However, estimates from these studies remain partial as they limited their analysis to enrolled parcels (avoided deforestation) and stakeholder perceptions. The reduced impact of PHS on environmental outcomes observed here and in other evaluations in Mexico, mirror a general trend observed in PES evaluations globally [18,64]. While most PHS evaluations rely on additionality and leakage [43,57], no robust evidence or consensus has emerged about targeting effectiveness of programs. Since literature shows that a focus on ES mapping can be useful in the context of PES [13,50,69], to our knowledge this study is one of the first efforts evaluating the targeting of PHS programs based on their ability to protect areas based on their potential capacity to provide HS.

The main focus of this paper was on spatially analyzing ES to quantify their relationships, identify areas of high provision, and clarify the biophysical factors involved. Then, we used this information to analyze the spatial overlaps between ES and zones targeted for PHS. We found that both eligible and payment zones had lower than expected coverage of predicted priority areas for individual (water yield, soil retention and carbon storage) and overlapping ES (as shown in Table 4 and Fig 5). Of particular relevance was the limited spatial overlap between these zones and priority areas for overlapping HS (water yield and soil retention), given how CONAFOR has sought to bundle these services [28] (see Table 5). Additionally, there was also a considerable lack of spatial congruence between the zones targeted by PHS and areas of high social importance (measured via marginalization) both when considered independently and combined with important areas for HS (see Fig 6). In general, there was a notable decline in the ability of PHS targeting zones to cover areas important ecologically (ES provisioning) or socially (marginalization) as these factors were combined or bundled.

Our study showed that clear synergies exist between studied ES and identified areas where the all ES could be enhanced simultaneously at the regional scale. This was possible due to spatial clustering and positive but relatively weak correlations between target and non-target ES. Considering correlation between ES as a measure of spatial similarity, carbon and soil retention were the most similar, followed by soil retention and water yield. However, the correlations between carbon storage and water yield were found weak. These findings mirror previous studies that have targeted areas providing multiple ES as a key component for effective decision-making in PES programs [7,18,24,40,70]. The majority of case studies using correlation coefficients reported similar relationships among pairs of ES and showed an increased ability to identify trade-offs [68]. In Mexico, this focus could enhance coordination between PHS programs and other emerging national programs such as REDD+. The moderate correlations we documented may result from differences in ecological drivers, including distribution of precipitation rates, and LULC [24,67,96]. These correlations suggest that strategies for conserving some HS (soil retention) but not others (water yield) could simultaneously improve provisioning of other ES such as carbon storage. Calls to identify and clarify synergies, and possible trade-offs between ES [45] to create bundles of services that can be targeted in PES are increasing in the literature [12–13,69–70]. However, given the observed relationship between water yield and soil retention, relying on HS as a single entry in payments without staking them is a barrier to improve program efficiency [21,42]. Staking of HS may be a useful strategy for program operators to highlight their importance for multiple user groups, increase their financial support and ability to include areas where additionality and opportunity costs are high [19,26]. As the context of each watershed is unique, program operators will need to evaluate the particular hydrological challenges and the groups of downstream beneficiaries to determine which particular HS should be targeted. Finally, program operators need to be aware of the fact that priority areas for maximizing service provisioning could shrink dramatically as more ES (including social priorities) are combined (see trends in Table 4).

Our results suggest an overall spatial mismatch between the zones targeted for PHS and most areas providing high levels of ES provision (see Tables 4 and 5). We believe this is a robust finding, since this pattern was documented for both individual and overlapping ES, at two spatial scales (eligible and payment zones), and for two levels of analysis (top 20 and 33% of pixels). Evaluated ES exhibited clear non-random spatial distributions, or clumping that probably contributed to these mismatches by reducing the probability that priority areas could be covered by PHS targeted zones under assumptions of random placement (see Tables 2 and 6). Also contributing to this trend was our focus on HS as the main evaluation criteria and CONAFOR’s focus on dense and threatened forest in the upper portions of the subwatersheds, since 2007, to help define eligible zones [30,57]. Despite being mostly forested, analyses and mapping of HS showed that provisioning was moderate in these areas, characterized by steep slopes, high rates of evapotranspiration and less precipitation than the regional annual average (see S1 and S2 Figs). These factors underpin HS supply [66–67] and led to lower levels of modeled water yield and soil retention. HS provisioning was also favored by and spatially associated with more intensified areas of lower vegetation density such as riparian vegetation, shade coffee, and secondary forest (see Table 3). This pattern is supported by previous studies suggesting that forest cover may not maximize the provisioning of some HS [12,58,76,94]. For instance, while modeled priority areas provided 42,242 m^3^ of water per year per ha, provision in eligible and payment zones was lower (see Table 7B). Given the lack of HS baselines, the national PHS program often fails to provide financial support to the most important areas for such services. Which represents an important challenge to program operators in seeking to achieve their ecological goals. These findings highlights the potential utility of a service-based spatial prioritization for stablishing HS baselines, improving PHS targeting, and guaranteeing HS provision. Spatial HS assessments and tools deserve more attention as they foster more informed decisions and ensure the long-term capacity of PHS targeted zones to provide HS [97].

Although the PHS targeted zones were not ideal in terms of complete overlap with HS, they covered important areas for carbon storage (see Tables 4 and 5) but provided services less efficiently that predicted priority areas, as shown in Table 7. This is due to (1) an overemphasis on forests in delimiting eligible zones for PHS, as well as (2) a lack of knowledge about the contribution of other land uses that may be important for HS delivery (see Table 3). The discordance we observed between forests and HS delivery highlights the limits of using this cover as a surrogate for HS delivery and supports previous studies criticizing this assumption as too simplistic [33,37–38,98]. Although Mexico’s national PHS program has been relatively understudied [30–32], numerous studies elsewhere have documented that PHS program impacts may be limited as they largely ignore the biophysical attributes of the landscape and forest cover that are critical for HS [5,99–101]. Given the complexity of the relationships between ecological and hydrological processes supporting HS, program operators may risk counterintuitive results when relying on forests [12,21,65,99].

Tools for spatially modelling ES such those used in this study have been shown to be effective means for evaluating PHS [48,69,102] and thus making more informed decisions to target payments within watersheds. Nevertheless, many PHS programs suffer from a paucity of local data and opt to evaluate their performance at national scale [22,45]. This scale is far from being operational since site-specific data is needed at the watershed scale to adequately model relevant hydrological processes [13,50,103]. Our subwatershed-scale analyses also highlight the critical need to include criteria and indicators directly linked to HS in program performance and evaluations [34,43,51]. In applying this idea, program operators should quantify the provision of HS via long-term monitoring, thus ensuring that providers actually maintain and restore the HS paid for [63]. While providing yet another way to evaluate programs, evidence of their ability in securing the provision of target ES is limited and has been highlighted as the most elusive component in evaluations [15–17]. Measures of the environmental outcomes of PHS programs in Mexico and elsewhere, are often complicated [94–95] and compounded by the poor understanding of how land uses contribute to HS provisioning [38,68,86]. Consequently, PHS are rarely tied directly to water-related outcomes, instead relying on proxies that may undermine their impacts [18,34]. Our findings support this conclusion and suggest that, by explicitly considering provision and the distribution of priority areas for HS as a main targeting criteria in PHS programs should improve their effectiveness.

Another key challenge for improving the targeting of payments is the identification of areas that could potentially help meet contrasting objectives [17–18]. While we primarily aimed at testing the extent to which PHS directly contribute to HS provisioning, findings from this study suggest that spatial prioritization can be useful in testing the hypothesis that PHS can serve both as a conservation and development instrument [31,64]. The simple methodology used in this study suggest that it is possible to identify areas that aim at providing multiple ES, based on ES co-occurrences, while also tackling marginalization (Fig 6). However, we found that the PHS targeted zones had lower success in covering these areas, resulting in 7.9% of overlap. This support the idea that PHS effectiveness may be negatively affected by the inclusion of secondary objectives such as poverty alleviation [23,68,104–105]. While achieving both social and socioeconomic goals holds great appeal, evidence suggests limited effects of PES on socioeconomic wellbeing [34,57,61]. Most studies show PES/PHS programs fail to adequately target areas of high deforestation pressure while also generating marginally positive effects on poverty alleviation [30,43,51,95,105]. Studies in Mexico suggest that over time the national PHS program has increased its focus on socioeconomic rather than environmental goals [52–53,62], which may weaken PHS impacts. Given the results presented here and the lack of convincing evidence that such goals can be simultaneously reached, PHS operators should try to refine the goals of their programs using well identified and monitored criteria, which will provide clear evidence of targeting performance. Here we provided a relatively simple approach to identify areas likely to provide multiple ES and target multiple benefits. However, ideally spatial optimization methods would help define solutions in which no particular objective can be improved without impairing at least one other objective [106–107]. Optimization and genetic algorithms, such as NSGII, have been used to investigate ES relationships and trade-offs between conflicting objectives [108]. Although, accounting for spatial information explicitly and determining optimal land use solution pose computational challenges, similar approaches could be used in the future to optimize ES provisioning, as they allow identify areas where the focus on HS can be in potential conflict with other objectives of the PHS program.

Decision-making tools have indeed the potential to influence PHS and decision-making. InVEST models proved to be valuable for considering underlying factors affecting ES, in understanding ES relationships, and for guiding decision-making in regards to the evaluation of PHS impacts. However, they are also known to be sensitive to biophysical and LULC patterns [48,80]. Part of the attraction of this tool is the rapid parameterization using a combination of data from multiple sources and spatial scales (Table 1). However, such flexibility has an inevitable degree of uncertainty and limits the accuracy of predictions [48–47]. Missing spatial data and scale issues were undoubtedly factors affecting model accuracy in this study. We used local data whenever possible in model parameterization. Nevertheless, on occasions we relied on information from studies in similar regions when site-specific observations for model parameters were missing (see S1 File). For example, model parameters for soil retention such as root depth, soil erodibility, and rainfall erosivity were derived by combining regional and national data. In the WY model, estimations of annual reference evapotranspiration were limited by inherent inaccuracies in national data sets. This model’s structure made seasonal variability impossible to estimate, thus highlighting the need for supplementary tools such as SWAT or Tier 2 InVEST models to further enhance model accuracy and utility when the necessary time and resources are available [44]. The carbon model is oversimplified and assumes fixed storage levels for each LULC type regardless of possible gains or losses with changes in structure, location, and microclimate. Future studies using this tool will undoubtedly benefit from including local and finer-scale field observations to validate model predictions [90]. In spite these limitations, we considered the InVEST models to be useful for our study, as they require relatively little data inputs and its products can inform decision related to ES. While studies based on the spatial rigor of ES with more complex algorithms (e.g. SWAT, LUCI, among others) could increase spatial detail in distribution of ES, they could led to significant increase in computing time, field monitoring and the need of high spatial resolution data. However, when modelling at a local scale high resolution spatial data may not be available. Our study is not intended for devising detailed management for each ES, but rather for helping identify areas where ES are spatially congruent, so effective strategies can be implemented to best deliver both ecological and social targets.

Additional limitations in our approach were that we did not include estimates of deforestation risk and HS demand, typically considered in the definition of PHS targeted areas in Mexico [28–32]. These factors could have influenced the distribution of modeled priority areas and their inclusion in future mapping efforts could improve applicability and accuracy to improve targeting [47,61]. Finally, we should mention that the threshold used here for defining priority areas was arbitrary, as in other studies [8,24,67], and thus does not provide a universal standard of areas that should be covered by PHS. Taken together these observations suggest that our predictions and findings should be viewed with some caution.

Despite above-mentioned limitations the models and analyses performed here are considered viable tools for first order approximations of ES provisioning in the literature [24,40,50,72], and are increasingly being used to detect important areas in conservation planning [56,70,108]. As decision makers are seeking tools to target investments, even simple analyses using models such as InVEST can facilitate understanding of ES patterns and underlying ecological processes to help inform decisions when more detailed data or monitoring of ES are lacking.

## Conclusions

This study used quantitative methods for spatially prioritizing ES, analyzing ES relationships, and identifying ES co-occurrences to evaluate the targeting effectiveness of Mexico’s National PHS program. While we demonstrate the need for more refined targeting methods, we also show that synergies exist between ES. We have illustrated the potential utility of spatial prioritization to help identify and target lands where desired HS can effectively be delivered. By using InVEST to prioritize areas for HS, our approach yielded results suggesting that program targeting may be poor in subwatersheds in central Veracruz. In particular, we found that zones targeted for PHS varied substantially in their spatial congruence with priority areas for maximizing ecological and social goals, and that this mismatch limits their potential impacts. While the identification of areas where targeted payments could help achieve both targets, local program operators will have to determine where this is possible and convenient. Despite the methodological limitations involved in the spatial mapping of ES, the potential benefits of such models for improving PHS performance should not be underestimated. In Mexico and elsewhere research focused on the mapping and documenting the spatial relationships between ES is scarce and needs more emphasis in the future. This, combined with long-term monitoring of PHS should help elucidate important drivers, and help improve model parameterization. Furthermore, should greatly advance our understanding of ES relationships and the design of the programs designed to conserve such services. Finally, future research is urgently needed to help clarify the impacts of PHS, and to better balance ecological and socioeconomic targeting criteria, to help programs maximize their performance and benefits.

## Supporting information

S1 File Spatial datasets and data inputs for the InVEST modeling. Complete description for mapping ecosystem services and manipulation of spatial data to generate all inputs for the InVEST’s Tiers 1 models.
(DOCX)

S1 Fig Spatial distribution of each model parameter used in InVEST. (a) Average root restricting depth values, (b) average annual precipitation, (c) average annual reference evapotranspiration, and (d) plant available water content values.
(TIF)

S2 Fig Spatial distribution of each model parameter used in InVEST. (a) Soil erodibility, (b) rainfall rodibility, (c) digital elevation model, and (d) the LS-slope-length factor.
(TIF)
