# Supplementary material for: Spatial analysis of ecosystem service relationships to improve targeting of payments for hydrological services
Source: PLoS One. 2018 Feb 20;13(2):e0192560. doi: 10.1371/journal.pone.0192560 (PMC5819813; doi:10.1371/journal.pone.0192560)
Supplement: S1 File — Complete description for mapping ecosystem services and manipulation of spatial data to generate all inputs for the InVEST’s Tiers 1 models. (DOCX) [file pone.0192560.s001.docx]

**Spatial analysis of ecosystem service relationships to improve targeting of payments for hydrological services.**

**Mokondoko et al.**

Pierre Mokondoko: División de Posgrado, Instituto de Ecología A.C. Xalapa 91070, Veracruz, México.

*Robert H. Manson: Red de Ecología Funcional, Instituto de Ecología A.C. Xalapa 91070, Veracruz, México.

Taylor Ricketts: Rubenstein School for Environment and Natural Resources; Gund Institute for Ecological Economics, University of Vermont, Burlington 05405, Vermont, USA.

Daniel Geissert: Red de Ecología Funcional, Instituto de Ecología A.C. Xalapa 91070, Veracruz, México.

*To whom correspondence should be addressed. E-mail: robert.manson@inecol.edu

This document includes:

Detailed description and accuracy of spatial of datasets and data inputs used in the InVEST modeling.

Figures S1 to S2

References

**Supporting Information**

**S1 File. Detailed description and accuracy of spatial datasets and data inputs used in the InVEST modeling.**

This document describes, both spatial and non-spatial, general data needs and data sources used to map two target (water yield and soil retention) and one non-target ES (carbon storage) in the spatially-based tool InVEST. We specifically used the water yield, sediment retention and carbon storage models for central Veracruz state. These models are based on simplifications of well-known biophysical relationships [1-2] and simple ecological production functions [3]. Models are parameterized on biophysical tables and explicit input layer that include land use/cover, topography, annual precipitation, annual reference evapotranspiration, soils, plant available water fraction, watersheds boundaries, etc. In this study, we carried out an exhaustive literature search, and analyzed data from 60 studies. Thus, to analyze distributions and spatial relationships between all ES we used InVEST version 3.3. Finally, all spatial layers were geo-referenced using WGS 84 Zone 14N datum and Universal Transverse Mercator (UTM) projection.

**Water yield model and input datasets:**

This model is based on the water balance principles and the Budyko curve [4-6]. It runs in a gridded map and estimates the amount of annual average precipitation that flows through the landscape minus storage, and evapotranspiration losses in each 20 m pixels [2,7]. For modeling the annual water yield, we parameterized this model using: average annual precipitation (mm y^-1^), average annual reference evapotranspiration (mm y^-1^), soil depth (mm), rooting depth (mm), plant available water content (fraction [0.1]), plant root depth (mm), subwatersheds boundaries, seasonality factor (fraction [0,1]) and land use/cover (LULC) attributes reflected in a biophysical table. The model provides tree types of outputs: rasters, providing information per pixel; shapes, summarizing pixel statistics at subwatershed level; and tables, providing information at the subwatershed scale [8]. As rasters reflect the spatial variability in LULC, they were the main inputs for the spatial analyzes in this study. In order to successfully run the model, following data inputs were used and estimated:

Land use/Land cover *(LULC)*: we used an 8-class GIS raster with the LULC types obtained from the SPOT images, which has a spatial resolution of 20m (see methodological section). Soil depth: a GIS raster was generated using the soil profiles from the national soil survey dataset named “*Serie II*” [9]. This dataset includes soil types, texture, type of soil horizons, particle composition, nutrient content, chemical and physical properties data. Thus, we estimated soil depth from 120 profiles distributed across the study area. With a linear regression analysis between total soil depth values and slope gradient obtained from a 15m digital elevation model (*r^2^* = 0.58; [9]), we then interpolated this relationship into a 20m × 20m raster. Annual precipitation (*Px*)**:** a GIS raster was performed using daily meteorological observations from 45 weather stations during 1966-2006, that were obtained from the ERIC III system [10]. Average annual precipitation values were generated using the IDW interpolation method in ArcGis v. 10.2. Plant available water content (*PAWC*): here defined as the difference in volumetric water content between field capacity and permanent wiling point [11-12]. Field capacity (*FC*) and wilting point (*PWP*) are parameters directly related to soil’s hydraulic conductivity and soil porosity [13-14]. Then they were predicted using soil texture (%sand, %clay and %silt) and organic matter content (OM) data from the national soil survey and the software “Soil Water Characteristics Tool” ([15]; <http://hydrolab.arsusda.gov/soilwater/Index.htm>). Finally, the map of the plant available water content was performed using the Kriging interpolation method in ArcGis 10.2 into a 20m raster. Annual reference evapotranspiration (*ETo*): defined as the energy supplied by the sun to vaporize water [2,8]. This parameter was calculated using daily mean, maximum and minimum temperature observations from the weather stations network, extraterrestrial radiation and the modified Hargreaves method [16-17], which generates accurate results when information is uncertain [18]. The map of annual potential evapotranspiration was obtained through the Kriging interpolation method into a 20m raster. Watersheds and sub-watersheds (*shp*): watersheds were obtained from the hydrographical network dataset [9] along with the map of subwatersheds of FIRCO [19], at 1: 250,000 scale and for each subwatershed one identification number was given. A biophysical table was performed to represent attributes of each LULC type, including LULC code, descriptive name of LULC, root depth and actual evapotranspiration. Root depth (*mm*): maximum root depth was obtained by identifying measured rooting depth of key vegetation types for each LUL when available [20-22]. When these values were missing, we used the Schenk and Jackon (2002) method [23], based on soil depth, where root biomass occurs in the 90% of the longest tap root (maximum depth). Rooting depth values for crops were found in FAO 56 guidelines [18,24]. Actual evapotranspiration coefficients (*ETk*): These coefficients are related to the characteristics of vegetation, and it is used to calculate annual potential evapotranspiration that controls the annual average water yield [25]. ETk values were obtained for each LULC types from Bruijnzeel et al. (2006) [26], Holwerda et al. (2010 and 2013) [27-28], and Muñoz-Villers et al. (2012) [29]. Finally, the seasonality coefficient Z (Zhang constant): which represents the seasonal rainfall distribution and depth precipitation [16], and was considered to be 4, at which results will have the least error [2].

Insert S1 Fig

**Sediment retention model and datasets**:

Here known as “soil retention model”. This model estimates the ability of land parcels (LULC) to retain soils (ton ha^-1^ yr^-1^) as a function of geomorphology, rainfall, topography, vegetation and management practices [1,3]. First, this model uses the Universal Soil Loss Equation (USLE) to calculate the average annual potential soil loss on every pixel [30] and then determines how much of these eroded soils may be retain downstream by different land uses; and finally the amount of soil reaching the streams [2,31]. Flow paths are calculated for water flowing after a precipitation events, thus, soils coming from upstream pixels are trapped at a given efficiency rate in a particular pixel and the amount that is not trapped going down in a combination with the soils eroded from that pixel [32]. Thus, it estimates how much soil reaches a stream and how much each pixels retains. For simulating soil loss, we analyzed data to estimate soil erodibility (*K factor*), rainfall erosivity (*R factor*), land use types (LULC), crop factor (*C*), management practice factor (*P*) and sediment retention efficiency (%). In order to run the model, following data inputs were used and estimated:

For *K* and *R* factors, while nonmanages land uses dominates very few data were available for the region and in the literature. Soil erodibility or K factor **(***ton·ha·* hr (MJ*·ha·mm*)^-1^**):** is a GIS raster representing the susceptibility of soils to erosion by rainfall events. We analyzed information on the soil physical properties from the national soil survey (n = 120) including soil structure, soil porosity, proportion of sand, silt and clay [9]. We then used the next equation proposed by Williams and Renard (1983) [3]:

$$K=\frac{\{0.2+0.3\exp\left[ -0.0256SD\left( 1-\frac{Si}{100} \right) \right]\}\times\left[ \frac{Si}{\left( Cl+Si \right)^{2}} \right]^{0.3}\times\{1-\frac{0.25}{\left[ C+\exp\left( 3.72-3.95C \right) \right]}\}\times[1-0.7\left( 1-\frac{\mathrm{Ds}}{100} \right)]}{\{1-\frac{Sd}{100}+exp[-5-51+22.9\left( 1-\frac{Sd}{100} \right)\}\}} (1)$$

Where Sd, Si, Cl and C represent percentage of sand, silt, and carbon, respectively. We used the Soil Water Characteristics Tool to estimate carbon (%). Then *K* values for each profile and soil types were estimated [34] and interpolated using the Kriging interpolation method to generate a 20m raster. ***Soil erosivity factor*** (*MJ·mm* (*ha·hr*)^-1^): a GIS raster that represents the intensity and duration of rainfall. Given the fact that we were not able to obtain rain intensity data, we followed the method proposed by Renard and Freimud (1994) [35], estimating annual erosivity as a function of a modified Fourier coefficient. This index was calculated using annual and monthly precipitation data obtained from the weather stations network to estimate a 20 m raster. Digital Elevation Model (*m*): a GIS raster derived from the contour lines of the topographic datasets for human-earth system from INEGI [9] at scale 1:50,000. The LS factor**:** slope length and slope steepness were computed using this MDE [9] and routed by the model using the method proposed in the Handbook 703 by the USDA [2,31]. The same watershed/subwatershed and LULC map described in previous section were used. Biophysical table: this table includes crop cover (*C*) and conservation support practices (*P*) factors and the soil retention coefficient in present vegetation (%). For C factor, we analyzed the values from the literature [36-38]. P values were set to 1 for the LULCs since management practices are not the focus of this study and nonmanaged land uses dominates, then we were not able to investigated such values. The last correspond to the retained amount of soils by each pixel as a function of the retention coefficient associated with each LULC type. The amount of soils retained from each pixel was obtained from Gupta and Larson (1979) [39], Calder (2002) [40], Pérez-Nieto et al. (2005) [41], Ponette-González et al. (2010) [36], Orué et al. (2011) [42], Rivera-Toral (2012) [37], the USLE manual [43] and the InVEST manual [2].

Insert Fig. 2 S1

**Carbon storage model and datasets:**

Based on a simplification of the carbon cycle, using maps of LULC types and data on stocks of the five carbon pools (aboveground biomass, belowground biomass, soil, dead organic matter and harvested wood products), this model estimates the amount of carbon (Mg C ha^-1^) currently stored in the landscape [1,3]. Thus, we used the same 8-class LULC map for the year 2010, described in previous sections as a baseline. However, estimating carbon storage for these LULC types was quite challenging, as data on carbon storage for the region was sparse, then we were only to obtain only estimates from four carbon pools. The carbon pool that was not considered in this study was carbon in harvested wood products. This procedure involved combining the current LULC raster with a table articulating carbon pool values by LULC types. For the different carbon pools *we*: collected data for aboveground and belowground biomass from the National Forest and Soil Inventory [44], Hughes et al. (1999) [45], Jaramillo et al. (2013) [46], Díaz et al. (2007) [47], Torres-Rivera et al. (2011) [48], Orihuela-Belmonte et al. (2013) [49], Cartus et al. (2014) [50], González-Molina et al. (2014) [51], and Gonzáles-Zamora et al. (2016) [52]. Estimates of carbon storage for shade coffee were found in Albrecht and Kandji (2003) [53], Hernández-Vázquez et al (2012) [54], Romero-Alvarado et al. (2002) [55] and Manson et al. (2008) [56]. Estimates of carbon storage for agricultural and managed grasslands were found in IPCC (2006) [57] and Hernandez et al. (2015) [58]. For soil carbon, we used soil organic, carbon content and bulk density data from the 120 profiles [9] to estimate carbon content for each profile and soil type, except for urban content. Also, data for soil carbon was found in Campos et al. (2007) [59], Campos et al. (2014) [60], Hernández-Vázquez et al (2012) [54] and Hernandez et al. (2015) [58]. In land use types for which no information was available, it was necessary to assign a value of zero.

**References**

1. Tallis, H., & Polasky, S. 2009. Mapping and valuing ecosystem services as an approach for conservation and natural-resource management. The year in ecology and conservation Biology 2009: *Ann. N. Y. Acad. Sci*., 1162: 265-283.
2. Tallis, H.T., Ricketts, T., Nelson, E., Ennaanay, D., Wolny, S., Olwero, N., et al. 2010. InVEST 1.004 beta User’s Guide. The Natural Capital Project, Stanford University <http://www.naturalcapitalproject.org/InVEST.html> (accessed 05.04.13).
3. Bhagabati, N. K., Ricketts, T., Sulistyawan, T. B. S., Conte, M., Ennaanay, D., Hadian, O. et al. 2014. Ecosystem services reinforce Sumatran tiger conservation in land use plans. *Biological Conservation*, 169: 147-156.
4. Zhang, L., Potter, N., Hickel, K., Zhang, Y., & Shao, Q. 2008. Water balance modeling over variable time scales based on the Budyko framework–Model development and testing. *Journal of Hydrology*, 360(1): 117-131.
5. Geng, X., Wang, X., Yan, H., Zhang, Q., & Jin, G. 2014. Land use/land cover change induced impacts on water supply service in the upper reach of Heihe River Basin. *Sustainability*, 7(1): 366-383.
6. Hamel, P., & Guswa, A. J. 2014. Uncertainty analysis of a spatially-explicit annual water-balance model: case study of the Cape Fear catchment, NC. *Hydrology & Earth System Sciences Discussions*, 11(9).
7. Vigerstol, K. L., & Aukema, J. E. 2011. A comparison of tools for modeling freshwater ecosystem services. *Journal of environmental management*, 92(10): 2403-2409.
8. Kareiva, P. 2011. Natural capital: theory and practice of mapping ecosystem services. *Oxford University Press*.
9. INEGI 2012. Instituto Nacional de Estadística y Geografía (INEGI). Conjuntos de datos Vectoriales Nacionales de Recursos Naturales.
10. IMTA (Instituto Mexicano de Tecnología del Agua). 2006. Extractor Rápido de Información Climatológica III, v. 1.0. Información climatológica disponible en formato electrónico. Veracruz, México.
11. Zhou, W., Liu, G., Pan, J., & Feng, X. 2005. Distribution of available soil water capacity in China. *Journal of Geographical Sciences*, 15(1): 3-12.
12. Tallis, H., Kareiva, P., Marvier, M., & Chang, A. 2008. An ecosystem services framework to support both practical conservation and economic development. *Proceedings of the National Academy of Sciences,* 105(28): 9457-9464.
13. Saxton, K. E., & Rawls, W. J. 2006. Soil water characteristic estimates by texture and organic matter for hydrologic solutions. *Soil science society of America Journal*, 70(5): 1569-1578.
14. Tranter, G., Minasny, B., McBratney, A. B., Murphy, B., McKenzie, N. J., Grundy, M., et al. 2007. Building and testing conceptual and empirical models for predicting soil bulk density. *Soil Use and Management*, 23(4): 437-443.
15. Saxton, K. E., Rawls, W., Romberger, J. S., & Papendick, R. I. 1986. Estimating generalized soil-water characteristics from texture. *Soil Science Society of America Journal*, 50(4): 1031-1036.
16. Zhang, L., Dawes, W. R., & Walker, G. R. 2001. Response of mean annual evapotranspiration to vegetation changes at catchment scale. Water resources research, 37(3): 701-708.
17. Leh, M. D., Matlock, M. D., Cummings, E. C., & Nalley, L. L. (2013). Quantifying and mapping multiple ecosystem services change in West Africa. *Agriculture, ecosystems & environment*, 165: 6-18.
18. Droogers, P., & Allen, R. G. 2002. Estimating reference evapotranspiration under inaccurate data conditions. *Irrigation and drainage systems*, 16(1): 33-45.
19. Fideicomiso de Riesgo Compartido [Firco]. 2006. Guía técnica para la elaboración de planes rectores de producción y conservación (PRPC). México D.F.: Secretaría de Agricultura, Ganadería, Desarrollo Rural, Pesca y Alimentación.
20. Canadell, J., Jackson, R. B., Ehleringer, J. B., Mooney, H. A., Sala, O. E. & Schulze, E. D. 1996. Maximum rooting depth of vegetation types at the global scale. *Oecologia*, 108(4): 583-595.
21. Andrade, J. L., Meinzer, F. C., Goldstein, G. & Schnitzer, S. A. 2005. Water uptake and transport in lianas and co-occurring trees of a seasonally dry tropical forest. *Trees*, 19(3): 282-289.
22. Masuhara, A., Valdés, E., Pérez, J., Gutiérrez, D., Vázquez, J. C., Pérez, E. S., & García, A. M. 2015. Carbono almacenado en diferentes sistemas agroforestales de café en Huatusco, Veracruz, México. *Revista Amazónica Ciencia y Tecnología*, 4(1), 66-93.
23. Schenk, H. J., & Jackson, R. B. 2002. Rooting depths, lateral root spreads and below‐ground/above‐ground allometries of plants in water‐limited ecosystems. *Journal of Ecology*, 90(3), 480-494.
24. Allen, R.G., Pereira, L.S., Raes, D. & Smith, M. 1998. “Crop evapotranspiration. Guidelines for computing crop water requirements.” *FAO Irrigation and Drainage* Paper 56. Food and Agriculture Organization of the United Nations, Rome, Italy. Available at: <http://www.fao.org/docrep/x0490e/x0490e00.htm>
25. Liu, W., Hong, Y., Khan, S. I., Huang, M., Vieux, B., Caliskan, S. et al. 2010. Actual evapotranspiration estimation for different land use and land cover in urban regions using Landsat 5 data. *Journal of Applied Remote Sensing*, 4(1): 041873.
26. Bruijnzeel, L. A., Burkard, R., Carvajal, A., Frumau, K. F. A., Köhler, L., Mulligan, M. et al. 2006. Hydrological impacts of converting tropical montane cloud forest to pasture, with initial reference to northern Costa Rica. DFID Project Report.
27. Holwerda, F., Bruijnzeel, L. A., Muñoz-Villers, L. E., Equihua, M., & Asbjornsen, H. 2010. Rainfall and cloud water interception in mature and secondary lower montane cloud forests of central Veracruz, Mexico. *Journal of Hydrology*, 384(1): 84-96.
28. Holwerda, F., Bruijnzeel, L. A., Barradas, V. L., & Cervantes, J. 2013. The water and energy exchange of a shaded coffee plantation in the lower montane cloud forest zone of central Veracruz, Mexico. *Agricultural and forest meteorology*, 173: 1-13.
29. Muñoz-Villers, L., F. Holwerda, M. Gomez-Cardenas, M. Equihua, H. Asbjornsen, L.A. Bruijnzeel, B.E. et al. 2012. Water balances of old growth and regenerating montane cloud forests in Central Veracruz, Mexico. *Journal of Hydrology* 462−463:53−66.
30. Wischmeier, W. H., & Smith, D. D. 1978. Predicting rainfall erosion losses-a guide to conservation planning. Predicting rainfall erosion losses-a guide to conservation planning.
31. Hamel, P., Chaplin-Kramer, R., Sim, S., & Mueller, C. 2015. A new approach to modeling the sediment retention service (InVEST 3.0): Case study of the Cape Fear catchment, North Carolina, USA. *Science of the Total Environment*, 524: 166-177.
32. Borselli, L., Cassi, P. & Torri, D. 2008. Prolegomena to sediment and flow connectivity in the landscape: a GIS and field numerical assessment. *Catena*, 75(3): 268-277.
33. Williams, J. R., Renard, K. G., & Dyke, P. T. 1983. EPIC: A new method for assessing erosion's effect on soil productivity*. Journal of Soil and water Conservation*, 38(5): 381-383.
34. Rahman, M. R., Shi, Z. H., & Chongfa, C. 2009. Soil erosion hazard evaluation: an integrated use of remote sensing, GIS and statistical approaches with biophysical parameters towards management strategies. *Ecological Modelling*, 220(13): 1724-1734.
35. Renard, K. G., & Freimund, J. R. 1994. Using monthly precipitation data to estimate the R-factor in the revised USLE. Journal of hydrology, 157(1-4): 287-306.
36. Ponette‐González, A. G., Weathers, K. C., & Curran, L. M. 2010. Water inputs across a tropical montane landscape in Veracruz, Mexico: synergistic effects of land cover, rain and fog seasonality, and interannual precipitation variability. *Global Change Biology*, 16(3): 946-963.
37. Rivera-Toral, F., Pérez-Nieto, S., Ibáñez-Castillo, L. A., & Hernández-Saucedo, F. R. 2012. Aplicabilidad del Modelo SWAT para la estimación de la erosión hídrica en las cuencas de México. *Agrociencia*, 46(2): 101-105.
38. Kumar, S., & Kushwaha, S. P. S. 2013. Modelling soil erosion risk based on RUSLE-3D using GIS in a Shivalik sub-watershed. *Journal of earth system science*, 122(2): 389-398.
39. Gupta, S., & Larson, W. E. 1979. Estimating soil water retention characteristics from particle size distribution, organic matter percent, and bulk density. *Water resources research*, 15(6): 1633-1635
40. Calder, I. R. 2002. Forests and hydrological services: reconciling public and science perceptions. Land Use and Water Resources Research, 2(2): 1-12.
41. Pérez-Nieto, J., Valdés-Velarde, E., Hernández-San Román, M. E., & Ordaz-Chaparro, V. 2005. Lluvia, escurrimiento superficial y erosión del suelo en sistemas agroforestales de café bajo sombra. *Agrociencia,* 39(4): 409-418.
42. Orúe, M. E., Booman, G. C., & Laterra, P. (2011). Uso de la tierra, configuración del paisaje y el filtrado de sedimentos y nutrientes por humedales y vegetación ribereña. Valoración de Servicios Ecosistémicos: Conceptos, Herramientas y Aplicaciones Para el Ordenamiento Territorial; INTA Ediciones: Buenos Aires, Argentina: 237-254.
43. Renard, K.G., Foster, G.R., Weesies, G.A., Porter, J.P., 1991. RUSLE. Revised Universal Soil Loss Equation. J. *Soil Water Conserv*. 46: 30–33
44. The National Forestry Commission of Mexico (CONAFOR). Inventario Nacional Forestal y de suelos. Informe 2013, del Estado de Veracruz (IEFyS). CONAFOR: Xalapa, México, 2014.
45. Hughes, R. F., Kauffman, J. B., & Jaramillo, V. J. 1999. Biomass, carbon, and nutrient dynamics of secondary forests in a humid tropical region of Mexico. *Ecology*, 80(6): 1892-1907.
46. Jaramillo, V. J., Ahedo-Hernández, R., & Kauffman, J. B. 2003. Root biomass and carbon in a tropical evergreen forest of Mexico: changes with secondary succession and forest conversion to pasture. *Journal of Tropical Ecology*, 19(04): 457-464.
47. Diaz, F. R., Acosta, M., Carillo, A., Buendía, R., Flores, A., & Etchevers, B. 2007. Determinación de ecuaciones alometricas para estimar biomasa y carbono en Pinnus patula Schl. et Cham. *Madera y Bosques*. 13(1): 25-34.
48. Torres-Rivera, J. A., Espinoza-Domínguez, W., Reddiar-Krishnamurthy, L., & Vázquez-Alarcón, A. 2011. Secuestro de carbono en potreros arbolados, potreros sin árboles y bosque caducifolio de Huatusco, Veracruz*. Tropical and Subtropical Agroecosystems*, 13(3): 543-549.
49. Orihuela-Belmonte, D. E., De Jong, B. H. J., Mendoza-Vega, J., Van der Wal, J., Paz-Pellat, F., Soto-Pinto, L., et al. 2013. Carbon stocks and accumulation rates in tropical secondary forests at the scale of community, landscape and forest type. *Agriculture, ecosystems & environment*, 171: 72-84.
50. Cartus, O., Kellndorfer, J., Walker, W., Franco, C., Bishop, J., Santos, L. et al. 2014. A national, detailed map of forest aboveground carbon stocks in Mexico. *Remote Sensing*, 6(6): 5559-5588.
51. González-Molina, L., Acosta-Mireles, M., Carrillo-Anzures, F., Báez-Pérez, A., & González-Camacho, J. M. 2014. Cambios de carbono orgánico del suelo bajo escenarios de cambio de uso de suelo en México. *Revista mexicana de ciencias agrícolas*, 5(7): 1275-1285.
52. González-Zamora, A., Esperón-Rodríguez, M., & Barradas, V. L. 2016. Mountain cloud forest and grown-shade coffee plantations: A comparison of tree biodiversity in central Veracruz, Mexico. *Forest Systems*, 25(1): 055.
53. Albrecht, A., & Kandji, S. T. 2003. Carbon sequestration in tropical agroforestry systems. *Agriculture, ecosystems & environment*, 99(1): 15-27.
54. Hernández-Vásquez, E., Ángeles, C., Virginia, G., Enríquez del Valle, J. R., Rodríguez-Ortiz, G., & Velasco Velasco, V. A. 2012. Captura de carbono por Inga jinicuil Schltdl: En un sistema agroforestal de café bajo sombra. *Revista mexicana de ciencias forestales,* 3(9): 11-21.
55. Romero-Alvarado, Y., Soto-Pinto, L., García-Barrios, L., & Barrera-Gaytán, J. F. 2002. Coffee yields and soil nutrients under the shades of Inga sp. vs. multiple species in Chiapas, Mexico. *Agroforestry Systems*, 54(3): 215-224.
56. Manson, R. H. 2008. Agroecosistemas cafetaleros de Veracruz: biodiversidad, manejo y conservación. Instituto de Ecología.
57. The Intergovernmental Panel on Climate Change (IPCC). 2006. 2006 IPCC Guidelines for National Greenhouse Gas Inventories, Volume 4: Agriculture, Forestry and Other Land Use. Prepared by the National Greenhouse Gas Inventories Programme, Eggleston, HS, L. Buendia, K. Miwa, T. Ngara, and K. Tanabe (eds). Institute for Global Environmental Strategies (IGES), Hayama, Japan. <http://www.ipcc-nggip.iges.or.jp/public/2006gl/vol4.html>.
58. Hernandez, M. E., Marín-Muñiz, J. L., Moreno-Casasola, P., & Vázquez, V. (2015). Comparing soil carbon pools and carbon gas fluxes in coastal forested wetlands and flooded grasslands in Veracruz, Mexico. International Journal of Biodiversity Science, Ecosystem Services & Management, 11(1), 5-16.
59. Campos, A., Oleschko, K., Etchevers, J., & Hidalgo, C. 2007. Exploring the effect of changes in land use on soil quality on the eastern slope of the Cofre de Perote Volcano (Mexico). Forest Ecology and Management, 248(3); 174-182.
60. Campos, A., Aguilar, G., & Landgrave, R. 2014. Soil organic carbon stocks in Veracruz State (Mexico) estimated using the 1: 250,000 soil database of INEGI: biophysical contributions. Journal of soils and sediments, 14(5): 860-87.

**List of Figures**

**Figure 1.** Spatial distribution of each model parameter used in InVEST (Part A)

**Figure 2**. Spatial distribution of each model parameter used in InVEST (continuation)
