# Supplementary figures and images for: Spatial analysis of ecosystem service relationships to improve targeting of payments for hydrological services
Source: PLoS One. 2018 Feb 20;13(2):e0192560. doi: 10.1371/journal.pone.0192560 (PMC5819813; doi:10.1371/journal.pone.0192560)

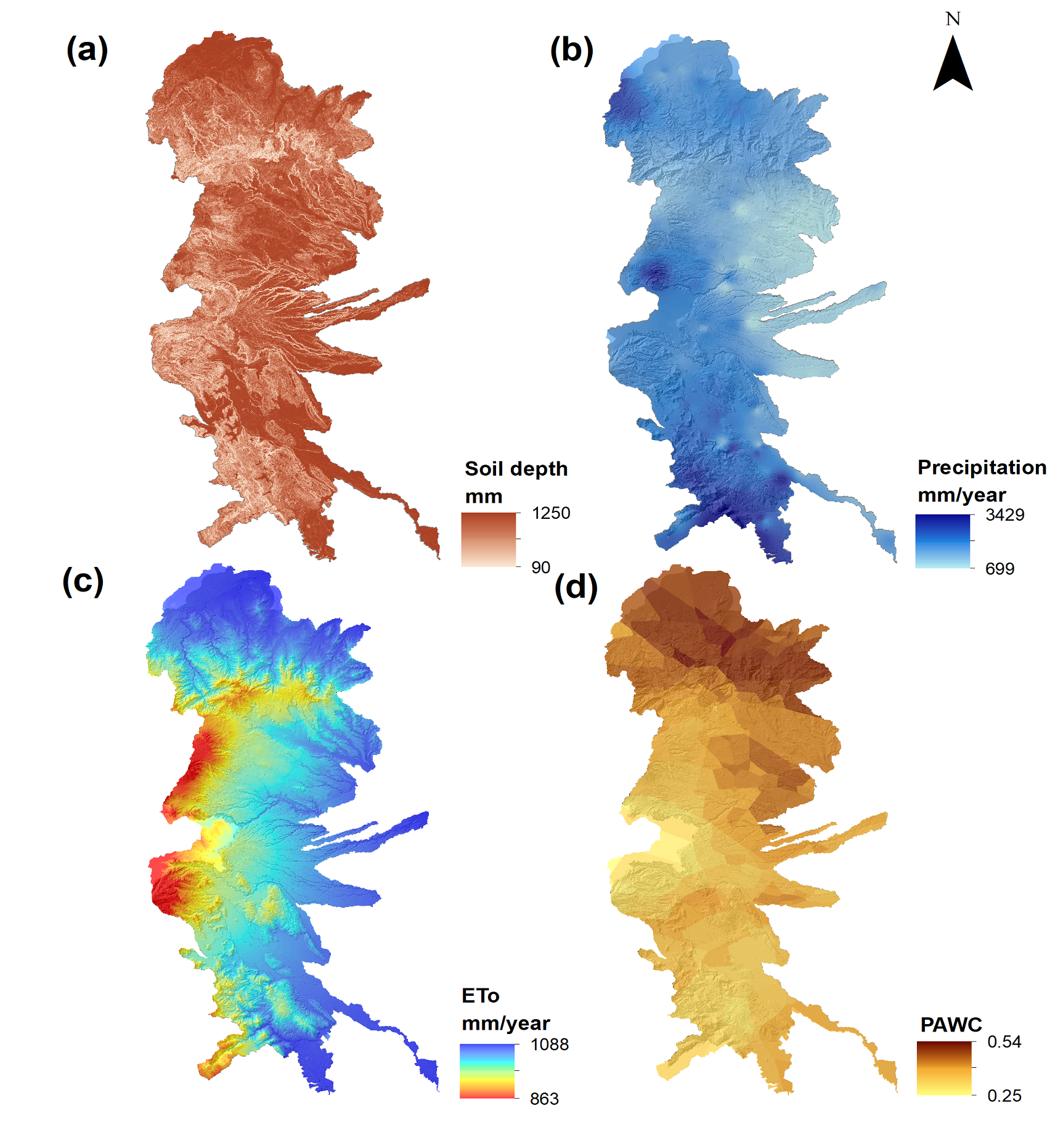

Supplement: S1 Fig — (a) Average root restricting depth values, (b) average annual precipitation, (c) average annual reference evapotranspiration, and (d) plant available water content values. (TIF) [file pone.0192560.s002.tif]

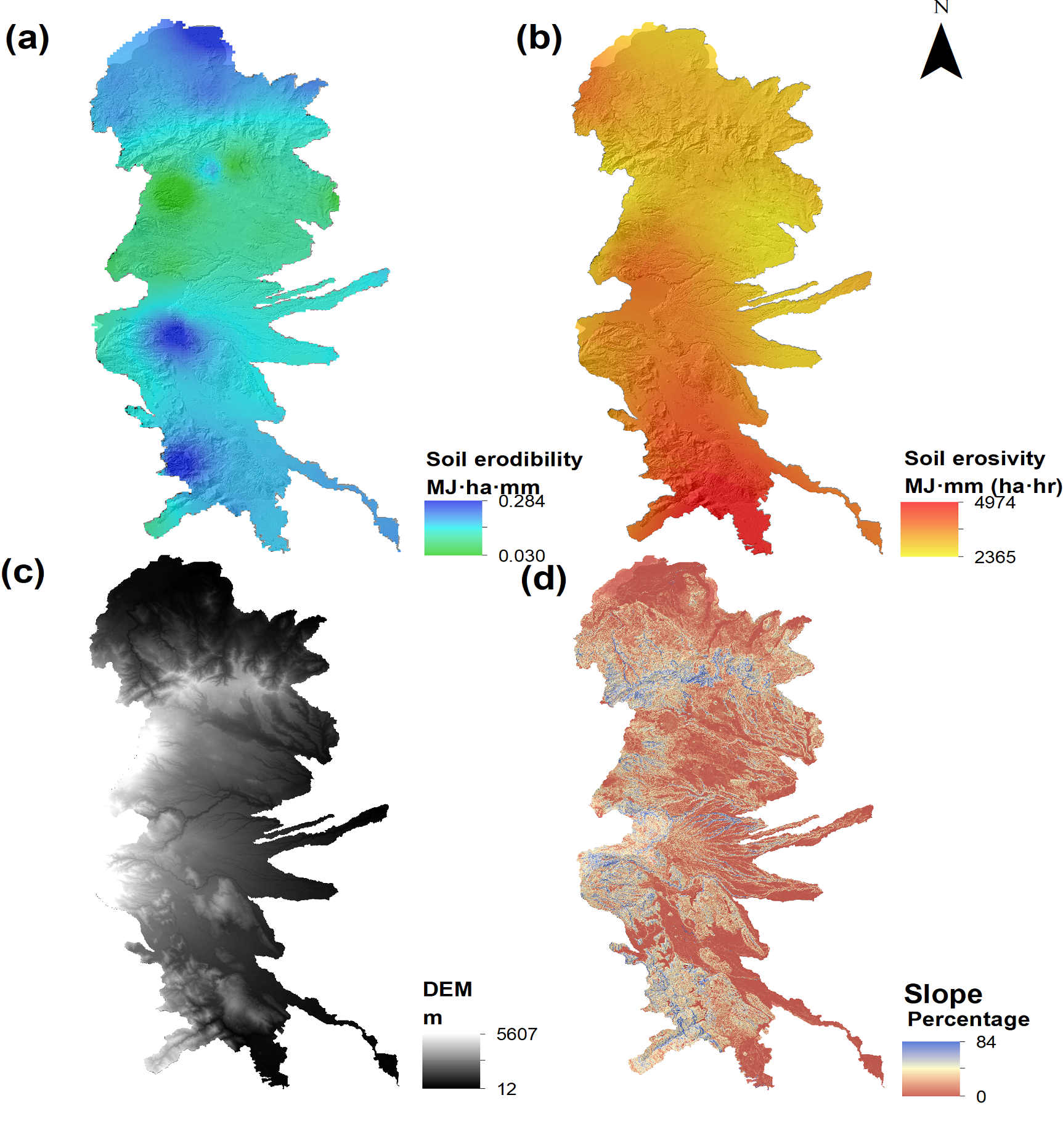

Supplement: S2 Fig — (a) Soil erodibility, (b) rainfall rodibility, (c) digital elevation model, and (d) the LS-slope-length factor. (TIF) [file pone.0192560.s003.tif]
